# Information needs and sources of information among people with depression and anxiety: a scoping review

**DOI:** 10.1186/s12888-022-04146-0

**Published:** 2022-07-27

**Authors:** Frederick H. F. Chan, Xiaowen Lin, Konstadina Griva, Mythily Subramaniam, Ivan Ćelić, Lorainne Tudor Car

**Affiliations:** 1 Lee Kong Chian School of Medicine, Nanyang Technological University, Clinical Sciences Building, 11 Mandalay Road, 308232 Singapore, Singapore; 2 Research Division, Institute of Mental Health, Singapore, SG Singapore; 3 University Psychiatric Hospital Vrapče, Bolnička cesta 32, HR-10090 Zagreb, Croatia; 4 Department of Primary Care and Public Health, Imperial College London School of Public Health, London, UK

**Keywords:** Information need, Information source, Information seeking, Depression, Anxiety, Scoping review

## Abstract

**Background:**

Previous studies have identified substantial unmet information needs in people with depression and anxiety. Sufficient information about the disorder, treatment, available services, and strategies for self-management is essential as it may influence quality of care and patients’ quality of life. This scoping review aimed to provide a broad overview of information needs of people with depression and anxiety as well as the sources that they use to seek this information.

**Methods:**

We included all primary research published in English that investigated information needs or information sources in people with depression or anxiety, with no restrictions imposed on the study design, location, setting, or participant characteristics. Six electronic databases (MEDLINE, Embase, PsycINFO, CINAHL, LISTA, Web of Science) and the grey literature (Google and Google Scholar) were searched for relevant studies published up to November 2021. Two reviewers independently screened articles and extracted data. Narrative synthesis was performed to identify key themes of information needs and information sources. Factors associated with information needs/sources such as demographic variables and symptom severity were also identified.

**Results:**

Fifty-six studies (comprising 8320 participants) were included. Information needs were categorised into seven themes, including general facts, treatment, lived experience, healthcare services, coping, financial/legal, and other information. The most frequently reported needs in both people with depression and anxiety were general facts and treatment information. Subclinical samples who self-reported depressive/anxious symptoms appeared less interested in treatment information than patients with clinical diagnoses. Information sources were summarised into five categories: health professionals, written materials, media, interpersonal interactions, and organisational resources. Health professionals and media (including the internet) were the most frequently adopted and preferred sources. Although few studies have examined factors associated with information needs and information sources, there is preliminary evidence that symptom severity and disease subtypes are related to information needs/sources, whereas findings on demographic factors were mixed.

**Conclusions:**

Information needs appear to be high in people with depression and anxiety. Future research should examine differences between subgroups and associated factors such as the treatment course. Personalised information provision strategies are also needed to customise information according to individual needs and patient profiles.

**Trial Registration:**

The protocol of this scoping review was registered on Open Science Framework (OSF; link: 10.17605/OSF.IO/DF2M6).

**Supplementary Information:**

The online version contains supplementary material available at 10.1186/s12888-022-04146-0.

## Background

Depressive and anxiety disorders are widespread across the world and are among the most common psychiatric disorders. In 2021, the estimated global prevalence of depression and anxiety were 3153 and 4802 cases per 100,000 population, respectively [1]. Depression and anxiety are highly comorbid with each other and are associated with increased mortality risk [2, 3]. Both conditions are considered as leading causes of disability worldwide and are major contributors to the global disease burden [4]. In 2020, COVID-19 pandemic has led to an additional substantial increase in the prevalence of depressive and anxiety disorders [1], which is expected to further add to the burden of disease even beyond the pandemic. However, few people with depression and anxiety seek mental health services, and in those who initiate treatment, many fail to receive follow-up care [5].

One of the explanations for this low rate of mental health service use may be the lack of information on the symptoms, diagnoses, and course and severity of the conditions, as well as the lack of information on available healthcare services and treatment options [6, 7]. Sufficient health information is essential in promoting patients’ self-management, preventing symptom exacerbation, informing treatment decisions, and improving treatment effectiveness [8]. On the other hand, patients’ ability to obtain, understand and use health information also plays an important role in high-quality care of mental disorders. A patient with high health literacy skills would be more likely than a patient with low health literacy to have adequate knowledge and skills to obtain and process information of their conditions and treatment options, and have the capacity to engage with the healthcare teams, navigate healthcare systems and optimise self-care of their conditions [9]. In brief, the provision of appropriate health information and patients’ actions to access and engage with this information are two key components of high-quality mental health care.

The Wilson’s model of information behaviour describes that information seeking behaviour arises as a result of information needs perceived by an individual who makes demands upon different information sources, which may lead to success or failure in retrieving relevant information [10, 11]. There are several subfunctions of information seeking, including requests for new information, requests for clarification, and requests for confirmation of known information [12]. To date, a fair amount of research has been conducted to explore the need for mental health information in people experiencing depression and anxiety, as well as the information sources that these individuals use or prefer to satisfy their needs. Many of these studies have highlighted substantial unmet information needs in people with depression and anxiety [7, 13–18]. This may be due to various factors including the lack of information about depression and anxiety provided to patients and the public, patients’ difficulties interpreting the provided information, information failing to reach the targeted population, or presentation formats not aligning with patients’ preferences. Indeed, some studies reported that some patients who are in contact with mental health services may still fail to absorb information provided by healthcare professionals [19] and may seek additional information from other sources such as the internet [16, 20–22].

As the global prevalence of depression and anxiety in the general population continues to grow [4, 23], there is a need to advance our understanding of information needs in people with depression and anxiety. It is also important to identify sources of information that these individuals have used as well as their preferences for the formats of information delivery. This improved understanding will enable more patient-centred mental health care that addresses important information needs in this population and will allow for more effective health communication strategies.

Several relevant reviews are available in the literature. However, these reviews mostly focused on needs for mental health care (i.e., needs for treatment, counselling, etc.) [24] and help seeking behaviour [25]. One systematic review was conducted on the information and decision-making needs of people with a range of mental disorders, but it was published nearly 10 years ago, without identifying any relevant studies on anxiety disorders, and did not review evidence regarding sources of information [14].

As such, using a scoping review methodology, we present a broad overview of the information needs and sources of information of people living with depression and anxiety. More specifically, we aimed to (1) determine the frequency of each information need as reported in the literature, (2) synthesise evidence regarding the types of current information sources and preferences for information sources, and (3) identify factors associated with information needs and information sources. Finally, we outline the research gaps in the current literature, future research recommendations as well as recommendations for provision of information to people with depression and anxiety. With this body of work synthesised, the current review would be able to guide the development of future information provision interventions targeted at depressive and anxiety disorders.

## Methods

### Protocol and registration

The protocol of this scoping review was registered on Open Science Framework (OSF; link: 10.17605/OSF.IO/DF2M6). We followed the JBI methodology of systematic scoping reviews [26]. We reported the findings in line with the Preferred Reporting Items for Systematic Reviews and Meta-Analyses: Extension for Scoping Reviews (PRISMA-ScR) [27].

### Eligibility criteria

We included all primary research published in English that investigated information needs or information sources in people with depression or anxiety, with no restrictions imposed on the study design, year of publication, study location, setting, or participant characteristics. Information needs refer to a perceived gap in a specific area of knowledge (e.g., treatment options for depression, etc.) which may result in information seeking behaviour [10]. In the current review, information needs can be either assessed by questionnaires asking participants if they are satisfied with the information given or if they would like to receive information on a certain topic, or can be identified through qualitative interviews. Information sources refer to the wide range of formats through which information is delivered (e.g., books, websites, etc.). In the current review, we differentiate between current information sources, which refer to sources individuals have used in the past or sources they are currently using, and preferred information sources, which refer to individuals’ preferences for the information format or how they would like to receive information in the future. In terms of the population, we included studies on patients diagnosed with depressive or anxiety disorders as well as studies on subclinical samples where participants self-reported high levels of depressive/anxious symptoms.

Studies that identified unmet information needs but did not specify the types of these needs were excluded. Studies that assessed general needs such as healthcare services need without mentioning specific information needs were also excluded. Moreover, studies that only reported needs for non-mental health information (e.g., information about COVID-19) were excluded. Studies were deemed irrelevant if they focused on patients with physical illness (e.g., cancer) who reported depressive/anxious symptoms because the presence of other medical conditions may affect patient demographics, and the information needs in these patients may differ from those with primary depression or anxiety. In addition, we excluded studies that focused on perspectives of family members, friends or caregivers of patients, as well as studies that involved only healthcare professionals (e.g., nurses, doctors).

### Search strategy

The following databases were searched from inception to 24 Nov 2021: MEDLINE, Embase, PsycINFO, CINAHL, Web of Science, and LISTA. We also searched the reference lists of included studies and any relevant systematic reviews to ensure all relevant articles were included. A search of grey literature through Google and Google Scholar was also performed. For the grey literature search, the first 10 pages of search results were used as 10 pages were deemed to be a good balance between the breadth of search and time needed for screening.

We selected keywords that were used frequently in titles/abstracts of relevant papers in this field and also consulted similar reviews for potential search terms. Subject headings (e.g., MeSH terms) as well as keywords in the title and abstract were searched. The search terms included exact words or synonyms of: information needs, information seeking behaviour, information sources, information search, internet search, online search, depression, and anxiety. We used the “explode” function and included all subheadings for the subject headings in order to capture relevant concepts and subtypes of depression and anxiety. The search strategy has been reviewed by the librarians at our university, and is presented in Table S1.

### Selection of sources of evidence

Titles and abstracts of all references retrieved from the search were independently screened by two reviewers (FC & XL) using EndNote. All studies were assessed for eligibility according to the specified inclusion and exclusion criteria. Disagreements between the two reviewers were resolved by discussion with a third reviewer (LTC). Irrelevant articles were excluded and the remaining studies were proceeded to full-text screening. All full-text articles were screened independently by the two reviewers to confirm relevance to this scoping review, and any discrepancies were resolved by discussion with the third reviewer.

### Data charting process

Data from the included studies were extracted to a Microsoft Excel spreadsheet. The information extracted included: author; year of publication; study location; study design and data collection methods; participant characteristics; type, severity and duration since diagnosis; study objective; types of information needs; types of current information sources; and types of preferred sources of information. Data extraction was performed independently by two reviewers (FC & XL), and disagreements were settled through discussion with the third reviewer (LTC). Study authors were contacted if there was any missing or incomplete information.

### Synthesis of results

We performed a narrative synthesis of the findings to identify key themes and subthemes of information needs and information sources. We used both a deductive and an inductive approach to form the themes and sub-themes. For information needs, we first adopted a framework covering six broad categories of information needs identified by previous research [13, 14, 28, 29], including (1) general facts about depression/anxiety, (2) treatment-related information, (3) lived experience, (4) healthcare service-related information, (5) coping and self-management, and (6) financial and legal information. Individual study findings were fit into these themes deductively and were further categorised into sub-themes inductively. An additional miscellaneous theme named “other information needs” was included during this process since some needs did not fit into any of the predetermined categories. In terms of current and preferred information sources, we adopted the typology developed by a similar review on cancer patients [28], with five themes including (1) health professionals, (2) written materials (e.g., books, leaflets), (3) media (e.g., internet, television, online forums), (4) interpersonal (e.g., friends, support groups), and (5) organisational resources (e.g., telephone services). Data were fit into these themes deductively, followed by inductive development of sub-themes. In this review, we reported the frequency of times each type of information needs or sources was mentioned in the literature. In addition, where possible we also attempted to synthesise findings on the relationships between study/participant characteristics (e.g., subtypes of depression/anxiety, gender, age, etc.) and information needs or information sources.

## Results

### Selection of sources of evidence

**Fig. 1:**
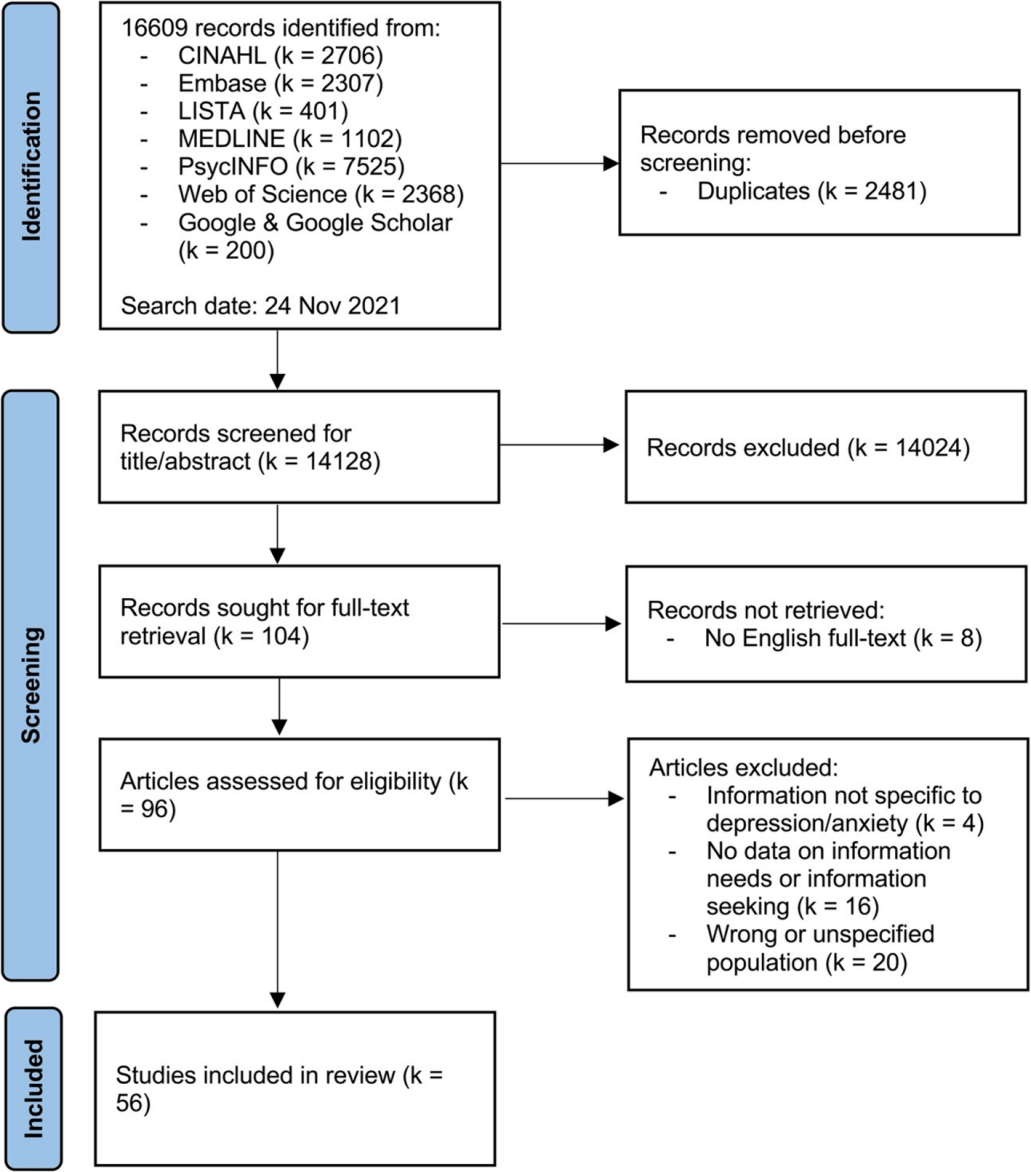
PRISMA flow diagram illustrating the search process

We identified a total of 16,609 records in our literature search. After removing 2481 duplicates, 14,128 articles were screened for titles and abstracts, after which the full-texts of 96 articles were retrieved for further screening. From these 96 articles, we excluded 40 studies that did not meet our inclusion criteria. Eventually 56 studies were included in this scoping review. Inter-rater reliability was excellent (kappa = 0.821). A PRISMA flow chart is presented in Fig. 1 [27].

### Characteristics of sources of evidence

Table 1 provides a summary of the characteristics of included studies. More detailed information of each included study is presented in Table S2. The 56 studies comprised of 31 quantitative studies, 20 qualitative studies, and five mixed-methods studies with 8320 participants. The majority of studies were conducted in high-income, western countries, including the US (k = 13), UK (k = 11), Germany (k = 9), Canada (k = 4) and Australia (k = 3). Sample sizes ranged from seven to 1288. Fifty-two studies included patients with depression or people who self-reported depressive symptoms. Twenty-four studies included patients with anxiety or people with self-reported anxiety symptoms. Forty-nine studies identified information needs in various categories, whereas 48 reported participants’ current sources of information and only 13 reported preferred sources of information.

**Table 1:** Summary of study characteristics

Domain	Study Characteristics	No. (%) of Studies (N = 56)
Study Design	Cross-sectional (quantitative, qualitative, or mixed-method)	51 (91.1%)
	Experimental (RCT, sequential control, etc.)	3 (5.4%)
	Cohort (prospective, longitudinal, etc.)	2 (3.6%)
Data Collection Methods	Quantitative	31 (55.4%)
	Qualitative	20 (35.7%)
	Mixed-methods	5 (8.9%)
Location (by country)	United States	13 (23.2%)
	United Kingdom	11 (19.6%)
	Germany	9 (16.1%)
	Canada	4 (7.1%)
	Australia	3 (5.4%)
	South Africa	2 (3.6%)
	Others	14 (25.0%)
Sample size	0–50	19 (33.9%)
	51–100	17 (30.4%)
	101–500	16 (28.6%)
	> 500	4 (7.1%)
Mean age	18–30	3 (5.4%)
	30–40	8 (14.3%)
	40–50	11 (19.6%)
	> 50	6 (10.7%)
	Others (i.e., unreported)	28 (50.0%)
Mental health conditions	Depression only	32 (57.1%)
	Anxiety only	4 (7.1%)
	Depression and anxiety	20 (35.7%)
Severity of conditions	Clinical diagnosis	39 (69.6%)
	Subclinical (self-reported symptoms)	17 (30.4%)

### Information needs

**Fig. 2:**
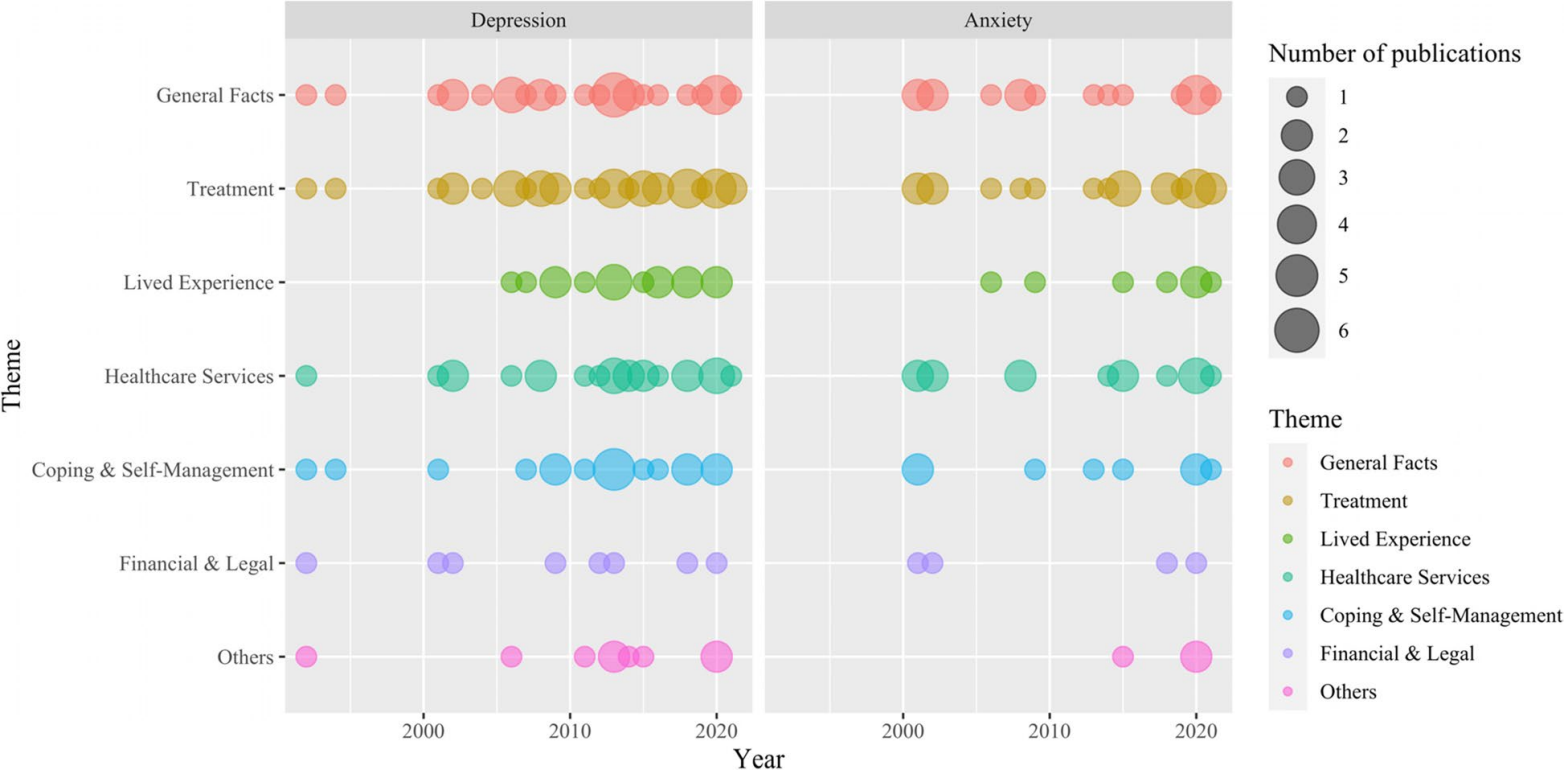
Publication frequency, by year, of included studies reporting each theme of information needs

We categorised information needs into seven broad categories, including (1) general facts about depression/anxiety, (2) treatment-related information, (3) lived experience, (4) healthcare service-related information, (5) coping and self-management, (6) financial and legal information, and (7) other information needs. These categories included 59 specific information needs for people with depression (Table 2 & Table S3) and 42 specific information needs for people with anxiety (Table 3 & Table S4). Information related to treatment was the most commonly identified theme for both depressive and anxious participants, and was also the theme with the most diverse number of specific information needs. Figure 2 presents the number of studies published over time in relation to each theme of information needs for people with depression and anxiety separately. Lived experience has only started to be mentioned by the literature after 2005, whereas other information themes have all been identified prior to this year.

**Table 2:** Information needs reported by studies on people with depression (k = 46 studies)

Theme	Information needs	Number and percentage of studies
General facts about depression (k = 32; 69.6%)	Symptoms/signs of depression	15 (46.9%)
	General information on depression	13 (40.6%)
	Diagnosis (diagnostic criteria; meaning of diagnosis)	11 (34.4%)
	Aetiology (causes of depression; scientific details)	10 (31.3%)
	Prognosis (length/course of depression; recovery)	6 (18.8%)
	Information on suicidal thoughts	4 (12.5%)
	Prevalence of depression	3 (9.4%)
	Recent research on depression	2 (6.3%)
	Whether depression is normal	2 (6.3%)
	Environmental risk factors (e.g., stress, lifestyle)	2 (6.3%)
	Risks of developing depression based on family history	2 (6.3%)
	Behavioural problems (e.g., violence, drug/alcohol abuse)	1 (3.1%)
Treatment (k = 38; 82.6%)	Treatment options and comparison between options	24 (63.2%)
	Side effects of treatment	19 (50.0%)
	Effectiveness/benefits of treatment and expected outcomes	15 (39.5%)
	General information on medication	14 (36.8%)
	Appropriate use of medication (e.g., dosage, long-term use, discontinuation)	13 (34.2%)
	Length of treatment	7 (18.4%)
	Explanation of specific procedures and approaches used	4 (10.5%)
	Issues related to addiction, tolerance, and dependence of medication	4 (10.5%)
	Adverse drug reactions	4 (10.5%)
	Psychosocial or nonpharmacological interventions	3 (7.9%)
	Mechanisms or (non-)pharmacological actions of medications	2 (5.3%)
	Placebo/nocebo effects	1 (2.6%)
	What does the prescribed dose indicate about patients’ condition	1 (2.6%)
Lived experience (k = 15; 32.6%)	Other people’s experience of depression in general	10 (66.7%)
	Other people’s experience of taking medications for depression	4 (26.7%)
	Other people’s experience of recovery from depression	2 (13.3%)
	Other people’s experience of antidepressant withdrawal and depression relapse	1 (6.7%)
	Other people’s reasons to use antidepressants	1 (6.7%)
	Previous patients’ experience of treatment	1 (6.7%)
Healthcare services (k = 24; 52.2%)	Available local services (e.g., hospitals, day treatment, rehabilitation)	14 (58.3%)
	How or where to get help	8 (33.3%)
	Support groups & patient associations	7 (29.2%)
	Healthcare professionals (e.g., psychologists, psychiatrists)	6 (25.0%)
	Information on the mental health system	2 (8.3%)
	Role of psychologists	2 (8.3%)
	How to get a further supply of medication	1 (4.2%)
Coping & self-management (k = 17; 37.0%)	Strategies to cope with depression and alleviate symptoms	13 (76.5%)
	Improving independent living skills (coping with everyday life)	5 (29.4%)
	Managing medication side effects	4 (23.5%)
	Improving social relationships and communication skills	3 (17.6%)
	Coping with stigma/discrimination	3 (17.6%)
	Strategies to speed recovery or prevent exacerbation	2 (11.8%)
	Coping with unpredictable variations in intensity and duration of depression	1 (5.9%)
	Strategies for solving problems	1 (5.9%)
	Dealing with weight gain	1 (5.9%)
	What to do in case of no response to medication	1 (5.9%)
Financial & legal information (k = 8; 17.4%)	Financial assistance	7 (87.5%)
	Cost of treatment	2 (25.0%)
	Mental health law	1 (12.5%)
Other information needs (k = 9; 19.6%)	Current health condition and other comorbid health problems	2 (22.2%)
	Social relationships and social isolation/avoidance/withdrawal	2 (22.2%)
	How to figure out severity of mental disorder	2 (22.2%)
	Interpretation of information from patient information leaflets and the internet	1 (11.1%)
	What to do between visits	1 (11.1%)
	Information for relatives	1 (11.1%)
	Work-related challenges	1 (11.1%)
	How to reduce the likelihood of depression for themselves and their family	1 (11.1%)

**Table 3:** Information needs reported by studies on people with anxiety (k = 23 studies)

Theme	Information Needs	Number and Percentage of Studies
General facts about anxiety (k = 17; 73.9%)	Symptoms	9 (52.9%)
	Diagnosis (diagnostic criteria; meaning of diagnosis)	7 (41.2%)
	Aetiology (causes of anxiety; scientific details)	5 (29.4%)
	General information on anxiety	3 (17.6%)
	Prevalence of anxiety	3 (17.6%)
	Prognosis (length/course of anxiety; recovery)	3 (17.6%)
	Whether anxiety is normal	2 (11.8%)
	Information on suicidal thoughts	2 (11.8%)
	Risks of developing anxiety based on family history	1 (5.9%)
Treatment (k = 21; 91.3%)	Treatment options and comparison between options	12 (57.1%)
	Side effects of treatment	11 (52.4%)
	General information on medication	9 (42.9%)
	Effectiveness/benefits of treatment and expected outcomes	7 (33.3%)
	Length of treatment	4 (19.0%)
	Psychosocial or nonpharmacological interventions	4 (19.0%)
	Appropriate use of medication (e.g., dosage)	4 (19.0%)
	Mechanisms or (non-)pharmacological actions of medications	3 (14.3%)
	Explanation of specific procedures and approaches used	2 (9.5%)
	Issues related to addiction, tolerance, and dependence of medication	2 (9.5%)
	Adverse drug reactions	2 (9.5%)
	What does the prescribed dose indicate about patients’ condition	1 (4.8%)
	Placebo/nocebo effects	1 (4.8%)
Lived experience (k = 7; 30.4%)	Other people’s experience of anxiety in general	6 (85.7%)
	Other people’s experience of taking medications for anxiety	1 (14.3%)
	Other people’s experience of recovery from anxiety	1 (14.3%)
Healthcare services (k = 14; 60.9%)	Available local services (e.g., hospitals, clinics, day care)	11 (78.6%)
	Healthcare professionals (e.g., psychologists, psychiatrists)	6 (42.9%)
	How or where to get help	5 (35.7%)
	Support groups & patient associations	5 (35.7%)
	Role of psychologists	1 (7.1%)
Coping & self-management (k = 8; 34.8%)	Strategies to cope with anxiety and alleviate symptoms	7 (87.5%)
	Improving independent living skills (coping with everyday life)	3 (37.5%)
	Managing medication side effects	1 (12.5%)
	What to do in case of no response to medication	1 (12.5%)
	Strategies to improve self-esteem	1 (12.5%)
	Coping with stigma/discrimination	1 (12.5%)
Financial & legal information (k = 3; 13.0%)	Financial assistance	2 (66.7%)
	Cost of treatment	1 (33.3%)
	Mental health law	1 (33.3%)
Other information needs (k = 4; 17.4%)	Information for relatives	2 (50.0%)
	How to figure out severity of mental disorder	1 (25.0%)
	Interpretation of information from patient information leaflets and the internet	1 (25.0%)

### Information needs of people with depression

Thirty-eight studies identified information needs related to treatment for depression. The top three information needs within this theme were treatment options and comparison between options (24 studies, 63.2%) [6, 13, 15, 16, 18, 20, 21, 30–46], side effects of treatment (19 studies, 50.0%) [6, 13, 16, 18, 21, 32, 34, 35, 38, 41, 43–45, 47–52], and effectiveness/benefits of treatments and expected outcomes (15 studies, 39.5%) [6, 13, 16, 18–21, 33, 34, 40, 41, 44, 49, 50, 53]. Other information needs included general information on medication (14 studies, 36.8%) [15, 16, 18, 20, 31, 35, 37, 44, 45, 47, 53–56], appropriate use of medication (13 studies, 34.2%) [13, 16, 19, 20, 22, 37, 41, 43, 48, 49, 51, 53, 57], length of treatment (7 studies, 18.4%) [19, 22, 33, 34, 41, 44, 49], explanation of specific procedures and approaches used (4 studies, 10.5%) [21, 41, 53, 58], issues related to addiction, tolerance, and dependence of medication (4 studies, 10.5%) [13, 19, 41, 48], adverse drug reactions (4 studies, 10.5%) [19, 20, 22, 41], psychosocial or nonpharmacological interventions (3 studies, 7.9%) [30, 49, 54], mechanisms of (non-)pharmacological actions of medications (2 studies, 5.3%) [41, 51], placebo/nocebo effects [51], and what the prescribed medication indicates about patients’ condition [22].

Thirty-two studies identified information needs for general facts about depression. Within these studies, the most frequently identified specific needs were symptoms/signs of depression (15 studies, 46.9%) [7, 15, 16, 18, 30, 35–37, 42, 45, 47, 50, 59–61], general information on depression (13 studies, 40.6%) [15, 16, 18, 30, 39, 44, 49, 52, 56, 59, 61–63], diagnosis (11 studies, 34.4%) [13, 21, 31, 32, 34, 35, 38, 45, 47, 55, 58], and aetiology (10 studies, 31.3%) [6, 13, 16, 18, 31, 35, 37, 41, 47, 61]. Other information needs within this theme included prognosis of depression (6 studies, 18.8%) [6, 16, 31, 36, 49, 58], information on suicidal thoughts (4 studies, 12.5%) [16, 37, 42, 64], prevalence of depression (3 studies, 9.4%) [18, 35, 47], recent research on depression (2 studies, 6.3%) [35, 61], whether depression is normal (2 studies, 6.3%) [34, 47], environmental risk factors of depression (2 studies, 6.3%) [35, 61], risks of developing depression based on family history (2 studies, 6.3%) [35, 61], and information on behavioural problems such as violence and alcohol abuse [35].

Twenty-four studies reported information needs within the theme of healthcare services, which included available local services and treatment facilities (14 studies, 58.3%) [6, 15, 16, 18, 30, 32, 33, 35, 45, 55, 56, 58, 59, 64], how or where to get help (8 studies, 33.3%) [13, 33, 38, 52, 54, 63–65], support groups and patient associations (7 studies, 29.2%) [16, 31, 32, 55, 56, 59, 64], healthcare professionals (6 studies, 25.0%) [6, 15, 16, 30, 56, 64], information on the mental health system (2 studies, 8.3%) [13, 35], role of psychologists (2 studies, 8.3%) [16, 34], and how to get a further supply of medication [49].

Seventeen studies identified information needs related to coping and self-management strategies. The top information need within this category was strategies to cope with depression and alleviate symptoms (13 study, 76.5%) [6, 13, 18, 30, 31, 35–37, 39, 45, 47, 59, 63]. Other coping-related information needs included improving independent living skills to cope with everyday life (5 studies, 29.4%) [6, 35, 37, 39, 41], managing medication side effects (4 studies, 23.5%) [20, 41, 49, 66], improving social relationship and communication skills (3 studies, 17.6%) [13, 35, 36], coping with stigma and discrimination (3 studies, 17.6%) [13, 18, 35], strategies to speed recovery or prevent exacerbation of depression (2 studies, 11.8%) [31, 49], coping with unpredictable variations in intensity and duration of depression [13], strategies for solving problems [35], dealing with weight gain [35], and what to do in case of no response to medication [41].

Fifteen studies reported information needs related to lived experience of depression. This theme included the following six sub-themes: other people’s experience of depression in general (10 studies, 66.7%) [6, 15, 18, 21, 30, 31, 33, 39, 43, 59]; other people’s experience of taking medications for depression (4 studies, 26.7%) [13, 20, 41, 44]; other people’s experience of recovery from depression (2 studies, 13.3%) [13, 21]; other people’s experience of antidepressant withdrawal and depression relapse [48]; other people’s reasons to use antidepressants [20]; and previous patients’ experience of treatment [43].

Eight studies identified needs for financial and legal information and seven (87.5%) reported information needs related to financial assistance for treatment [20, 31, 33–35, 52, 55]. In addition, two studies reported information need for cost of treatment [18, 20], and another study reported information need for mental health law [55]. Finally, nine studies reported information needs that could not be categorised into the abovementioned themes. In particular, the need for information on patients’ current health condition and comorbid health problems was identified in two studies [13, 44]. Some studies reported a lack of knowledge in people with depression regarding how to figure out severity of mental disorder [13, 18], and how to interpret information from patient leaflets and the internet [22]. Some studies also identified information needs related to specific aspects of daily life such as social relationships [35, 37] and work-related challenges [37]. In addition, people with depression requested information on what to do between visits [58], information for relatives [6], and how to reduce the likelihood of depression for themselves and their family [61].

### Information needs of people with anxiety

Twenty-one studies reported information needs related to the treatment for anxiety. The top cited information needs within this theme were also similar to those identified in people with depression, which were treatment options and comparison between options (12 studies, 57.1%) [7, 15, 17, 18, 21, 32–34, 41, 42, 45, 46], side effects of treatment (11 studies, 52.4%) [7, 17, 18, 21, 32, 38, 41, 45, 47, 51, 67], general information on medication (9 studies, 42.9%) [7, 15, 18, 45, 47, 54–56, 67], and effectiveness/benefits of treatments and expected outcomes (7 studies, 33.3%) [7, 17, 18, 21, 33, 41, 67]. Other information needs included length of treatment (4 studies, 19.0%) [22, 33, 34, 41], psychosocial or nonpharmacological interventions (4 studies, 19.0%) [7, 18, 54, 67], appropriate use of medication (4 studies, 19.0%) [22, 41, 51, 57], mechanisms of medications (3 studies, 14.3%) [17, 41, 51], explanations of specific procedures and approaches used (2 studies, 9.5%) [21, 41], issues related to addiction, tolerance, and dependence of medication (2 studies, 9.5%) [17, 41], adverse drug reactions (2 studies, 9.5%) [22, 41], what the prescribed dose indicates about patients’ condition [22], and placebo/nocebo effects [51].

Seventeen studies identified needs related to general facts about anxiety. The most commonly identified specific needs in people with anxiety were similar to that of people with depression, which were symptoms (9 studies, 52.9%) [7, 15, 17, 18, 42, 45, 47, 60, 67], diagnosis (7 studies, 41.2%) [17, 21, 32, 38, 45, 47, 55], and aetiology (5 studies, 29.4%) [7, 17, 18, 41, 47]. Other information needs within this theme included general information on anxiety (3 studies, 17.6%) [15, 18, 56], prevalence of anxiety (3 studies, 17.6%) [18, 34, 47], prognosis of anxiety (3 studies, 17.6%) [7, 17, 34]. whether anxiety is normal (2 studies, 11.8%) [34, 47], information on suicidal thoughts (2 studies, 11.8%) [64], and risks of developing anxiety based on family history [17].

Fourteen studies reported information needs related to healthcare services, with the majority mentioning available local services and treatment facilities (11 studies, 78.6%) [7, 15, 17, 18, 32–34, 45, 55, 56, 67]. Other service-related information needs included healthcare professionals (6 studies, 42.9%) [7, 15, 17, 56, 64, 67], how or where to get help (5 studies, 35.7%) [17, 33, 38, 54, 64], support groups and patient associations (5 studies, 35.7%) [17, 32, 55, 56, 64], and role of psychologists [34].

Eight studies mentioned information needs related to coping and self-management, and the majority (7 studies, 87.5%) mentioned specific strategies to cope with anxiety and alleviate symptoms [7, 17, 18, 34, 45, 47, 67]. Other less frequently mentioned information needs were improving independent living skills and coping with everyday life (3 studies, 37.5%) [7, 17, 41], managing medication side effects [41], what to do in case of no response to medication [41], strategies to improve self-esteem [17], and coping with stigma and discrimination [18].

Only seven studies reported information needs related to lived experience in people with anxiety. Of these, six reported needs for other people’s experience of anxiety in general [7, 15, 17, 18, 21, 33], one reported needs for other people’s experience of taking medications for anxiety [41], and one reported needs for other people’s experience of recovery from anxiety [21]. Only three studies identified needs related to financial/legal information in people with anxiety, with two reporting information on financial assistance for treatment [33, 55], one mentioning cost of treatment [18], and one mentioning information need for mental health law [55]. Finally, four studies reported other information needs, including information for relatives [7, 17], how to figure out severity of mental disorder [18], and how to interpret information from patient leaflets and the internet [22].

### Current information sources

**Fig. 3:**
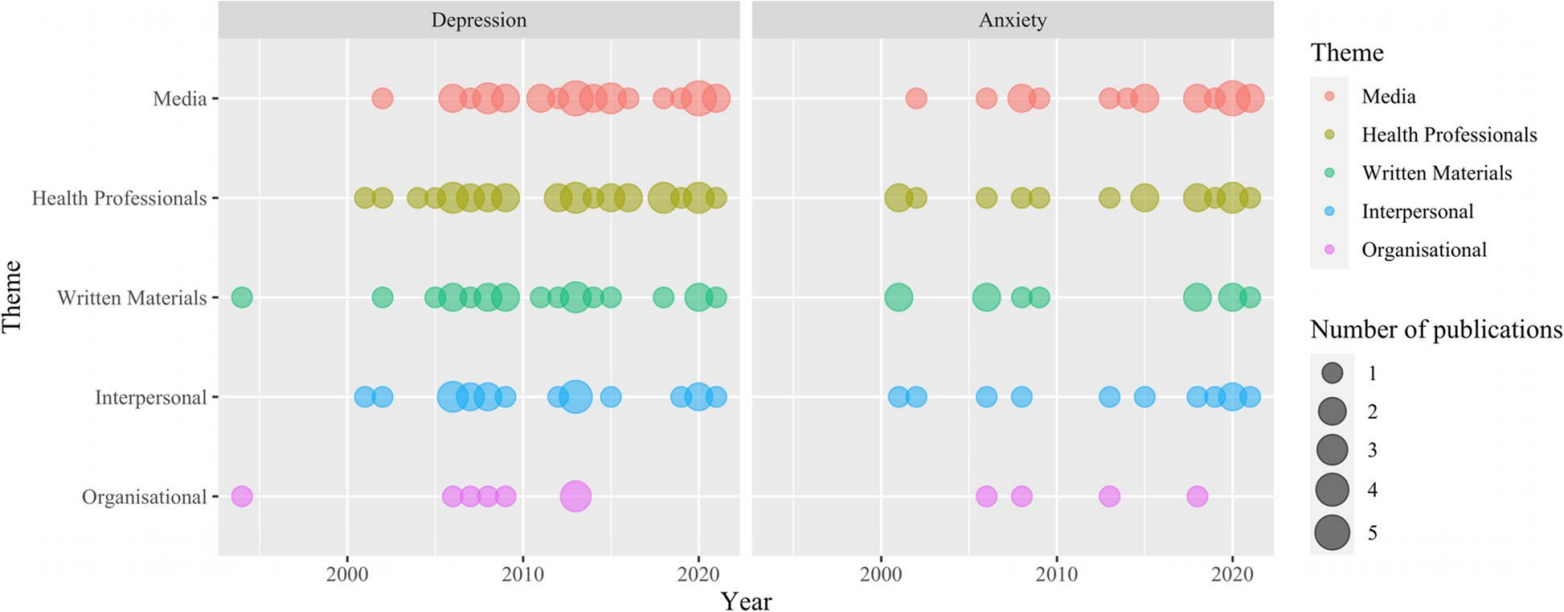
Publication frequency, by year, of included studies reporting each category of information sources

We categorised participants’ current information sources into five themes, namely health professionals (e.g., doctors, pharmacists), written materials (e.g., books, leaflets), media (e.g., internet, social media, broadcast media), interpersonal (e.g., talking to friends), and organisational resources (e.g., hotline). No additional theme emerged during the analysis. Figure 3 presents the number of studies published over time in relation to each category of information sources for people with depression and anxiety separately. It appears that written materials were mentioned more frequently as current information sources in earlier publications, whereas media resources were increasingly cited in more recent publications.

### Current information sources of people with depression

Current information sources of people with depression are summarised in Table S5. Media was the most frequently reported source of information (32 studies), including browsing the internet (e.g., depression websites, search engines, Wikipedia; 27 studies, 84.4%) [6, 15, 16, 18, 20–22, 30–32, 39, 41, 42, 44–47, 51, 52, 56, 57, 60, 61, 63, 64, 68, 69], broadcast media (11 studies, 34.4%) [31, 38, 39, 44, 47, 51–53, 68–70], and online forums/groups and social media (8 studies, 25.0%) [13, 15, 18, 20, 30, 37, 56, 57]. Health professionals was also a frequently mentioned information source (31 studies), including general healthcare professionals (26 studies, 83.9%) [6, 18, 20–22, 31, 33, 36, 38, 39, 41–44, 48, 50–53, 57, 60, 61, 70–73], mental health professionals (12 studies, 38.7%) [6, 18, 31, 33, 34, 38, 44, 47, 51, 53, 57, 64], pharmacists (6 studies, 19.4%) [20, 22, 41, 53, 57, 66], email communication with physicians [20], and email/chat or online appointments with mental health professionals [30].

Twenty-one studies reported written materials as an information source used by people with depression. Over half of these studies reported patient information leaflets, medicine labels, brochures/pamphlets (13 studies, 61.9%) [20–22, 31, 41, 44, 50–53, 68–70], and books (12 studies, 57.1%) [18, 21, 31, 36, 39, 44, 47, 61, 64, 68–70], as current information sources. Seven studies (33.3%) reported participants obtaining depression information from newspapers and magazines [31, 38, 39, 46, 51, 69, 70]. Twenty studies reported participants obtaining depression information from interpersonal interactions, including friends and relatives (15 studies, 75.0%) [18, 20, 31, 39, 42, 44, 47, 51, 53, 57, 60, 70–73], self-help or support groups (6 studies, 30.0%) [21, 31, 38, 59, 64, 67], emailing friends [20], and minister or spiritual advisors [18]. Finally, eight studies reported organisational resources including mental health organisations (3 studies, 37.5%) [21, 31, 64], general healthcare system and public sources (2 studies, 25.0%) [39, 44], telephone services and hotlines (2 studies, 25.0%) [20, 64], and classes on mental health (2 studies, 25.0%) [36, 47].

### Current information sources of people with anxiety

Current information sources of people with anxiety are summarised in Table S6. Media was also the most frequently cited source (19 studies), including browsing the internet (e.g., anxiety websites, search engines, Wikipedia; 18 studies, 94.7%) [7, 15, 17, 18, 21, 22, 32, 41, 42, 45–47, 51, 56, 57, 60, 64, 74], broadcast media (4 studies, 21.1%) [38, 47, 51, 74], and online forums or blogs (4 studies, 21.1%) [15, 18, 56, 57]. Health professionals was also commonly mentioned (16 studies), including general healthcare professionals (13 studies, 81.3%) [7, 18, 21, 22, 33, 38, 41, 42, 51, 57, 60, 67, 74], mental health professionals (10 studies, 62.5%) [7, 18, 33, 34, 38, 47, 51, 57, 64, 67], and pharmacists (3 studies, 18.8%) [22, 41, 57].

Eleven studies reported written materials as an information source, which included: patients information leaflets, medicine labels, brochures/pamphlets (5 studies, 45.5%) [21, 22, 41, 51, 67], newspapers and magazines (5 studies, 45.5%) [38, 46, 51, 67, 74], and books (5 studies, 45.5%) [18, 21, 47, 64, 74]. Eleven studies reported interpersonal interactions as a source of anxiety information, including friends and relatives (7 studies, 63.6%) [18, 42, 47, 51, 57, 60, 74], self-help or support groups (5 studies, 45.5%) [21, 38, 51, 64, 67], and ministers or spiritual advisors (2 studies, 18.2%) [18, 67]. Finally, four studies identified organisational resources, such as mental health organisations (2 studies, 50.0%) [21, 64], general healthcare system and public sources [74], telephone services and hotlines [64], and classes on mental health [47].

### Preferred information sources

Some included studies also identified participants’ preferred sources of information related to depression and anxiety. In people with depression (Table S7), information from health professionals was most preferred (10 studies). Eight (80.0%) of these studies reported general healthcare professionals as a preferred information source [20, 31, 42–44, 48, 61, 62], followed by mental health professionals [31, 61], pharmacists [20, 43], email communication with pharmacists [41], and online chat/appointments with experts [63]. Nine studies reported media as a preferred source of depression information, with seven (77.8%) reporting broadcast media [31, 40, 52, 59, 61–63], six (66.7%) reporting the internet [31, 40–42, 59, 61], and one reporting online forums and social media [63].

Six studies reported written materials as preferred depression information sources. These included patient information leaflets, brochures, and pamphlets (4 studies, 66.7%) [31, 40, 52, 59], books (4 studies, 66.7%) [20, 31, 40, 62], and magazines (2 studies, 33.3%) [40, 62]. Four studies mentioned organisational resources including educational outreach in hospitals, schools and churches (3 studies, 75.0%) [31, 42, 52], telephone services and hotlines [20], and informative signs or tables at community events [52]. Finally, only two studies reported interpersonal interactions as a preferred source of depression information, including friends and relatives [31, 42], and self-help or support groups [31].

In terms of studies involving participants with anxiety, only two reported preferred sources [41, 42]. Both studies reported browsing the internet and communication with health professionals as preferred sources of information. Communication with friend and relatives as well as organisational resources have also been mentioned by one of the studies [42].

### Variables linked to information needs and information sources

#### Demographics

Several studies investigated the relationship between demographic variables and information needs/sources, which revealed mixed results. Specifically, a quantitative study in India involving both patients with depression and anxiety found that male patients, and those with higher education levels, were more likely to seek information related to symptom identification, diagnostic criteria, and treatment options [45]. However, in another quantitative study conducted in Denmark involving only depression patients, gender was not associated with information needs [58]. Regarding information sources, a focus group study suggested that older participants with current/past depression diagnosis preferred books, physicians, pharmacists, and telephone services over the internet [20], whereas a quantitative study found no difference in where people looked for information based on gender, age, or race.

#### Comparison between conditions and severity levels

Based on the synthesised results, there appears to be no difference in information needs/sources between participants with depression and anxiety. In both groups, the most frequently requested information were treatment-related information (i.e., treatment options, medications, side effects, effectiveness and expected outcomes) and general disease-related information (i.e., symptoms, diagnosis, aetiology). Both groups also reported frequent use of media and health professionals as current sources of information. Comparison of preferred information sources was not possible due to the small number of studies reporting this outcome in the anxiety population.

Quantitative studies directly comparing information needs/sources between depression and anxiety are also lacking. One study found no difference in current information sources between patients with depression and patients with anxiety [42], while another study found that patients with anxiety were more likely to seek symptom-related information compared to those with depression [45].

We also performed analyses on the difference between severity levels of the conditions (i.e., clinical diagnosis vs. subclinical symptoms). Seventeen studies reported on subclinical samples who self-reported depressive or anxious symptoms [13, 18, 30, 52, 54, 55, 57, 60, 61, 63–65, 69, 71–74]. Within these studies, the most frequently mentioned needs were general disease-related information [13, 18, 30, 52, 55, 60, 61, 63, 64] and service-related information [13, 18, 30, 52, 54, 55, 63–65]. Treatment information need was less frequently mentioned (7 studies, 41.2%) [13, 18, 30, 52, 54, 55, 57]. This is different from the clinical population where treatment-related information was most needed. Also, although service-related information has been frequently requested in subclinical samples, these information needs were mainly concerned with general help-seeking (i.e., how or where to get help and support) rather than looking for available treatment facilities or health professionals. In terms of information sources, the pattern of usage of current sources appeared similar between clinical and subclinical populations, with Internet and health professionals being the most frequently used sources. However, the effect of symptom severity on preferred information sources cannot be confirmed due to the small number of studies reporting this outcome.

#### Subtypes of depression

The only disease subtype identified from the included studies was postpartum depression, with four studies reporting information needs/sources in this population [30, 59, 62, 63]. All four studies identified needs related to general facts about postpartum depression, including its symptoms and causes of symptoms. Three of these identified information needs related to healthcare services (i.e., available local service, support groups, healthcare professionals, and treatment facilities) and coping and self-management (i.e., strategies to cope with depression and address symptoms) [30, 59, 63]. Information needs related to lived experience (i.e., other people’s experience of depression) [30, 59] and treatment options [30] have also been mentioned but less frequently. No study reported needs for financial/legal information in this population.

Unlike the main analyses where treatment information was the most needed educational topic for people with depression, the subgroup analyses of people with postpartum depression showed that this may not be a priority compared to general facts about the condition, and information on available healthcare services and coping. Regarding information sources, none of the four studies mentioned written materials as a current information source, but two studies indicated that participants would like to receive information in such format [59, 62].

In addition, although depression with suicidal thoughts does not constitute a subtype of depression, one study compared information needs between depressive participants with and without suicidal intentions [55]. In particular, it was found that suicidal participants needed more information about social support groups, whereas non-suicidal participants wanted more information on diagnosis, medication, and financial/legal information [55].

## Discussion

### Summary of evidence

We identified 56 articles (8320 participants) investigating information needs and information sources of people with depression and anxiety. Most studies were conducted in high-income western countries. Treatment-related information was the most frequently identified information need in both participants with depression and anxiety, and was also the category with the most diverse information needs. Subgroup analyses showed that treatment information need may be more prevalent in patients with clinical diagnoses of depression/anxiety than subclinical participants who self-reported their symptoms.

In terms of information sources, media and health professionals were the two most commonly reported current sources for both depressive and anxious participants and did not seem to differ between clinical and subclinical samples. These two categories were also the most preferred information sources in people with depression. However, there is a lack of evidence of preferred information sources in people with anxiety. Moreover, although there is some evidence that information needs/sources may differ across age groups, gender, and disease subtypes, more research is needed to further confirm these associations.

Both participants with depression and anxiety reported high needs for general disease-related facts and treatment-related information. This finding is similar to an earlier systematic review of information needs in depression and schizophrenia [14]. Sufficient information on these two topics is essential because it is not only effective in enhancing patients’ understanding of the health problem, but also improving patients’ satisfaction, quality of life, communication with healthcare providers, and self-care behaviours (e.g., medication adherence) [8, 24]. Studies in other chronic disease populations have also found that patients who were given sufficient and accurate information are more likely to actively engage in treatment decision-making processes and have fewer decisional conflicts [8]. However, it is noteworthy that some participants in our included studies reported the fear that too much information may create excessive worry and anxiety, especially about medication taking [20, 53]. Some other patients also reported unwanted information such as medication side effects because they believed that such information would hinder their medication adherence or make them hypervigilant towards bodily symptoms [41]. Therefore, it may be important in clinical practice to use non-threatening language (e.g., avoiding medical terminology) to inform patients of the treatment and its side effects. It may also be helpful to educate patients about the nocebo effect. One experimental study included in this review reported that patient education on nocebo effect can in fact shift patients’ needs toward desiring less information on the potential side effects of antidepressants [51].

Taken together, these findings indicate that information provision should be tailored according to individual patients’ needs rather than following a one-size-fits-all approach [75]. Our finding that clinical samples may have higher needs for treatment information compared to subclinical samples also supports this suggestion to tailor the amount and type of information based on symptom severity, settings, and patient preferences. In addition, two studies reported that some participants did not know how to interpret depression and anxiety information provided on websites and in patient leaflets [13, 22]. This suggests that the complexity of information given to patients should also be adjusted according to their mental health literacy.

Besides basic facts and treatment information, around one third of the included studies identified information needs for other people’s lived experience of depression/anxiety, as well as strategies for coping and self-management. Interestingly, the “lived experience” information theme appeared to be an increasingly recognised need in recent years based on the publication trend. Indeed, some patients reported that hearing other people’s experience with similar problems is particularly helpful because it reassures them that they are not alone and instils hope that their depression/anxiety may recover [13, 21]. Similarly, learning from previous patients how they coped with symptoms and everyday lives may offer practical guidance for patients experiencing mental health conditions for the first time. These findings implicate that involving previous patients in the development of information resources may be of benefit. Quantitative and qualitative evidence have shown that peer support workers in mental health services (i.e., those who had similar experience of mental disorders) are able to promote hope in the possibility of recovery, increase self-esteem and self-efficacy, and improve self-management in patients currently experiencing mental disorders [76, 77]. A recent randomised controlled trial has also shown that a peer-delivered self-management intervention reduced readmission to acute care after a period of crisis team care [78]. Future research should determine the added value of including lived experience and self-management topics in information resources for depression and anxiety.

In terms of information sources, media was the most frequently used current source, and was also a frequently mentioned preferred source, in both people with depression and anxiety. There also appears to be an increase in the use of media for information-seeking based on the publication trend. Within the studies that reported media as the current source of information, the majority (> 80%) mentioned the internet such as using search engines and browsing depression and anxiety websites. With the rapid growth of information and communication technology, information-seeking behaviour has changed drastically over the past decades. Using the internet to seek health information has become a global trend due to its increased availability, convenience, coverage of information, anonymity, and interactivity [79–81]. These may be the reasons why internet has been reported as a more frequent source compared to written materials, interpersonal interactions, and organisational resources. The anonymity of the internet may be particularly helpful for people with depression and anxiety to seek information given the stigma associated with mental health conditions [21]. The rise of smartphone and social media in recent years may further increased online health information seeking [79]. Social media such as Facebook has become a heavily utilised source of information and a tool for self-education [82, 83]. Although in our analysis online communities and social media have only been mentioned by a small number of studies, this may be due to the heterogeneity across included studies regarding how information source was measured and reported. The majority of studies mentioned the internet as an information source, without further differentiating between various categories of online sources (e.g., organisational websites, Facebook, YouTube, Quora, etc.). There is a need for future research to investigate differences in information needs and information seeking between these various platforms given their distinct functions and unique user communities. For example, social media may be a source where information on lived experience is most sought for, whereas governmental websites may provide more information on general facts and available services.

It is noteworthy, however, that some participants in the current review reported not trusting web-based information and considered online discussion forums as mere entertainment for them [20]. Concerns have also been raised by these participants that online discussion forums may lead other people to misuse antidepressants [20]. Although these findings were reported more than 10 years ago and the quality of online information may have improved since then, a recent systematic review of 153 studies evaluating 11,785 health-related websites using the DISCERN scale reported that 37–79% of the website were of good quality, while the rest were of poor quality [84]. These suggest that efforts should be made to increase the trustworthiness of online resources. Depression and anxiety information should be consolidated on governmental websites or official websites of mental health organisations. One included study also mentioned online chat rooms moderated by experts as a preferred source of information [63].

The limitations of online information resources may be one reason why health professionals were cited as an equally important source of depression and anxiety information in the current review. Health information seekers may perceive healthcare professionals as a more trustworthy source that provides useful and relevant information compared to other sources [80]. However, our review found that general healthcare providers appeared to be a more frequently used and preferred source of information over mental health professionals for both people with depression and anxiety. This may be due to the lack of knowledge about or limited access to available mental health services, or may be due to the stigma associated with visiting a mental health professional. Future studies should seek to understand the barriers to and facilitators of mental health services use. Relatedly, there is a potential need to provide more mental health education and training for general practitioners in order for them to meet this need. Several qualitative studies have shown that although general healthcare providers value the need to integrate mental health care into their practice, they expressed concerns regarding their lack of knowledge about mental disorders, lack of clinical experience and lack of communication skills in treating these conditions [85–87]. A recent systematic review concluded that short mental health training courses for non-specialist healthcare providers improved knowledge, skills and confidence, as well as clinical practice and patient outcomes [88]. In addition, although pharmacists were not a frequently mentioned information source in this review, they may play a unique role in providing medication information to depression and anxiety patients, and therefore mental health training for this profession may also prove useful.

There is a lack of research on factors associated with information needs and information sources in people with depression and anxiety. Although some studies reported associations of information needs/sources with disease subtypes, age and gender, these studies were inconclusive and yielded mixed results. Investigating factors associated with information needs/sources is important because such research would advance our understanding of the different preferences of subgroups of patients, which would benefit future development of personalised information provision interventions. It has been suggested that patients given personalised information are more likely than patients who received general information to use it and feel that they have learnt new knowledge [8]. There have already been personalised information leaflets in other patient populations where the leaflets are customised according to patients’ profiles (e.g., underscoring certain items) and are updated at each visit [89, 90]. Similar work in the population of depression and anxiety may be a fruitful line of future research.

### Limitations

Several limitations of the current review warrant acknowledging. First, few studies have investigated factors associated with information needs/sources, and therefore these reported effects should only be seen as preliminary. Second, we only included English articles in this review and therefore may have missed relevant articles in other languages. Also, most included studies were cross-sectional. Future research in this field adopting longitudinal designs is needed to identify information needs at different stages or severity of depression and anxiety. It is also crucial to understand information needs at different stages of treatments (e.g., short-term vs. long-term users of antidepressants). Moreover, the current review only summarised the frequency of times that each information need or information source was mentioned in the literature. This is because of the methodological heterogeneity across studies in terms of how information needs/sources were quantified or reported. Therefore, findings of the current review should be interpreted with caution and should be confirmed by future research and systematic reviews. Another limitation is that we were not able to differentiate between various types of online information sources in the current review. In the majority of studies, authors reported the internet as a source as a whole, but did not further differentiate between different platforms. This precluded us from further analysis to examine differences in information behaviour across sites. Finally, only two studies reported preferred information sources in people with anxiety. Future research needs to investigate preferences of anxiety patients in terms of the presentation format of information provided to them.

## Conclusions

In conclusion, this scoping review synthesised evidence of information needs and information sources of people with depression and anxiety. Information needs included general disease-related facts, treatment, lived experience, healthcare services, coping, and financial/legal information. The most frequently mentioned needs were treatment information, general facts about the condition, and available healthcare services. These categories of information were similar between depression and anxiety and should be provided to both subclinical and clinical populations, with varying amount and type. Information sources were categorised into five themes, including health professionals, written materials, media, interpersonal, and organisational sources. Media and health professionals were the two most frequently used sources. These two sources were also the most preferred in people with depression, whereas there is a lack of research on preferred information sources in people with anxiety. There is preliminary evidence that information needs and sources may differ depending on symptoms severity, depression subtypes, and age and gender, but the small number of studies directly analysing these associations precludes us from a firm conclusion. Future research should aim to explore information needs at various stages of depression/anxiety and treatment course, determine patients’ preferred format and platform through which information is delivered, and attempt to develop personalised information provision strategies.

## Supplementary Information

**Additional file 1:  Table S1.** Search strategy (24 Nov 2021). **Table S2.** Overview of included studies. **Table S3.** Information needs reported by studies on people with depression (k = 46 studies). **Table S4.** Information needs reported by studies on people with anxiety (k = 23 studies). **Table S5.** Current information sources reported by studies on people with depression (k = 44 studies). **Table S6.** Current information sources reported by studies on people with anxiety (k = 22 studies). **Table S7.** Preferred information sources reported by studies on people with depression (k = 13 studies).

## Data Availability

The datasets used and/or analysed during the current study are available from the corresponding author on reasonable request.
