# Supplementary material for: Information needs and sources of information among people with depression and anxiety: a scoping review
Source: BMC Psychiatry. 2022 Jul 27;22:502. doi: 10.1186/s12888-022-04146-0 (PMC9326147; doi:10.1186/s12888-022-04146-0)
Supplement: Supplementary file 1 — Additional file 1: Table S1. Search strategy (24 Nov 2021). Table S2. Overview of included studies. Table S3. Information needs reported by studies on people with depression (k = 46 studies). Table S4. Information needs reported by studies on people with anxiety (k = 23 studies). Table S5. Current information sources reported by studies on people with depression (k = 44 studies). Table S6. Current information sources reported by studies on people with anxiety (k = 22 studies). Table S7. Preferred information sources reported by studies on people with depression (k = 13 studies). [file 12888_2022_4146_MOESM1_ESM.docx]

Table S1. *Search strategy (24 Nov 2021)*

| Database | S# | Search Terms | Results |
| --- | --- | --- | --- |
| CINAHL | 1 | (MH “Information Needs”) | 11,802 |
|  | 2 | (MH “Information Seeking Behavior”) | 4,771 |
|  | 3 | TI (information need* OR information seek*) OR AB (information need* OR information seek*) | 99,817 |
|  | 4 | TI (“information source*” OR “source* of information” OR “information search*” OR “search* for information” OR “online search*” OR “internet search*”) OR AB (“information source*” OR “source* of information” OR “information search*” OR “search* for information” OR “online search*” OR “internet search*”) | 11,496 |
|  | 5 | (MH “Depression+”) | 128,786 |
|  | 6 | (MH “Anxiety+”) OR (MH “Anxiety Disorders+”) | 99,483 |
|  | 7 | TI (depress* OR anxiety OR anxious) OR AB (depress* OR anxiety OR anxious) | 222,767 |
|  | 8 | (#1 OR #2 OR #3 OR #4) AND (#5 OR #6 OR #7)  Limiters – English Language; Exclude MEDLINE records | 2,706 |
| Embase (Ovid) | 1 | exp information seeking/ | 4,842 |
|  | 2 | (information need* or information seek*).mp. [mp=title, abstract, heading word, drug trade name, original title, device manufacturer, drug manufacturer, device trade name, keyword heading word, floating subheading word, candidate term word] | 16,602 |
|  | 3 | (“information source*” OR “source* of information” OR “information search*” OR “search* for information” OR “online search*” OR “internet search*”).mp. [mp=title, abstract, heading word, drug trade name, original title, device manufacturer, drug manufacturer, device trade name, keyword heading word, floating subheading word, candidate term word] | 38,532 |
|  | 4 | exp depression/ | 549,814 |
|  | 5 | exp anxiety disorder/ or exp anxiety/ | 494,253 |
|  | 6 | (depress* or anxiety or anxious).mp. [mp=title, abstract, heading word, drug trade name, original title, device manufacturer, drug manufacturer, device trade name, keyword heading word, floating subheading word, candidate term word] | 1,097,510 |
|  | 7 | (#1 OR #2 OR #3) AND (#4 OR #5 OR #6)  Limiter – English Language | 2,307 |
| LISTA | 1 | (DE “INFORMATION needs”) OR (DE “INFORMATION resources” OR DE “ARCHIVAL resources” OR DE “BIBLIOGRAPHY (Documentation)” OR DE “BIOGRAPHICAL sources” OR DE “ELECTRONIC information resources” OR DE “INFORMATION overload” OR DE “LIBRARY resources” OR DE “PHOTOGRAPHS as information resources” OR DE “PICTURES as information resources” OR DE “REFERENCE sources”) | 36,096 |
|  | 2 | TI (information need* OR information seek*) OR AB (information need* OR information seek*) | 41,215 |
|  | 3 | TI (“information source*” OR “source* of information” OR “information search*” OR “search* for information” OR “online search*” OR “internet search*”) OR AB (“information source*” OR “source* of information” OR “information search*” OR “search* for information” OR “online search*” OR “internet search*”) | 13,053 |
|  | 4 | TI (depress* OR anxiety OR anxious) OR AB (depress* OR anxiety OR anxious) | 5,265 |
|  | 5 | (#1 OR #2 OR #3) AND #4  Limiter – English Language | 401 |
| MEDLINE (PubMed) | 1 | “Information Seeking Behavior”[Mesh] | 3,055 |
|  | 2 | information need*[Title/Abstract] OR information seek*[Title/Abstract] | 11,789 |
|  | 3 | “information source*”[Title/Abstract] OR “source* of information”[Title/Abstract] OR “information search*”[Title/Abstract] OR “search* for information”[Title/Abstract] OR “online search*”[Title/Abstract] OR “internet search*”[Title/Abstract] | 11,814 |
|  | 4 | “Depression”[Mesh] OR “Depressive Disorder”[Mesh] OR “Anxiety”[Mesh] OR “Anxiety Disorders”[Mesh] | 367,523 |
|  | 5 | depress*[Title/Abstract] OR anxiety[Title/Abstract] OR anxious[Title/Abstract] | 649,584 |
|  | 6 | (#1 OR #2 OR #3) AND (#4 OR #5) | 1,102 |
| PsycINFO | 1 | DE “Information Seeking” OR DE “Questioning” | 7,312 |
|  | 2 | TI (information need* OR information seek*) OR AB (information need* OR information seek*) | 96,486 |
|  | 3 | TI (“information source*” OR “source* of information” OR “information search*” OR “search* for information” OR “online search*” OR “internet search*”) OR AB (“information source*” OR “source* of information” OR “information search*” OR “search* for information” OR “online search*” OR “internet search*”) | 15,221 |
|  | 4 | DE “Major Depression” OR DE “Anaclitic Depression” OR DE “Dysthymic Disorder” OR DE “Endogenous Depression” OR DE “Late Life Depression” OR DE “Postpartum Depression” OR DE “Reactive Depression” OR DE “Recurrent Depression” OR DE “Treatment Resistant Depression” OR DE “Depression (Emotion)” OR DE “Anxiety” OR DE “Anxiety Sensitivity” OR DE “Climate Anxiety” OR DE “Computer Anxiety” OR DE “Death Anxiety” OR DE “Health Anxiety” OR DE “Mathematics Anxiety” OR DE “Performance Anxiety” OR DE “Social Anxiety” OR DE “Speech Anxiety” OR DE “Test Anxiety” OR DE “Travel Anxiety” OR DE “Anxiety Disorders” OR DE “Castration Anxiety” OR DE “Generalized Anxiety Disorder” OR DE “Obsessive Compulsive Disorder” OR DE “Panic Attack” OR DE “Panic Disorder” OR DE “Phobias” OR DE “Separation Anxiety Disorder” OR DE “Trichotillomania” | 292,656 |
|  | 5 | TI (depress* OR anxiety OR anxious) OR AB (depress* OR anxiety OR anxious) | 464,119 |
|  | 6 | (#1 OR #2 OR #3) AND (#4 OR #5) | 7,525 |
| Web of Science | 1 | (“information need*” OR “information seek*” OR “information source*” OR “source* of information” OR “information search*” OR “search* for information” OR “online search*” OR “internet search*”) AND (“depress*” OR “anxiety” OR “anxious”) and English (Languages) | 2,368 |

Table S2. *Overview of included studies.*

| **Author, Year, Country** | **Study Design and Data Collection Methods** | **Participant** | **Gender** | **Age** | **Study Objective** |
| --- | --- | --- | --- | --- | --- |
| Al-Saffar et al., 2008, Kuwait | Randomised controlled trial (Questionnaire) | 150 newly diagnosed patients with unipolar depression and initiated with a single antidepressant; 79 (53%) participated in the scheduled follow-up after 6 weeks of treatment | Baseline: 45 female, 105 male; Follow-up: 19 female, 60 male | Range: 20-59 Mean: 34.5 SD: 9.5 | To assess patients’ opinion toward receiving written or specialised verbal pharmacists’ interventions and to determine the effect of these interventions on patients’ medication knowledge. |
| Anderson et al., 2013, UK | Qualitative (Interview) | 80 patients with depression taking antidepressants | 48 female, 25 male (7 not mentioned) | Range: 16-75 | To explore patient narratives interviews about depression and its treatment in order to improve patient and health professional understanding of what it is like to use antidepressants. |
| Balhara et al., 2020, India | Cross-sectional (Questionnaire) | 36 patients with mood disorders; 82 patients with anxiety disorders | N/A | N/A | To understand the pattern of problematic internet use among adult patients attending the outpatient psychiatry services at a tertiary care centre in India. |
| Barney et al., 2011, Australia | Qualitative (Analysis of online forum posts) | 134 participants with a current or a past history of depression | N/A | N/A | To investigate the explicit and implicit information needs of users of an online depression support forum. |
| Boath et al., 2004, UK | Qualitative (Written comments) | 60 women who had a baby aged between 6 weeks and 1 year and who had clinical depression | 60 female | Mean: 27.7 SD: 4.8 | To understand the role of specialist and routine care in postnatal depression treatment and pinpoint which aspects of specialist care are important to women |
| Boath et al., 2013, UK | Qualitative (Interview) | 15 first-time mothers with an infant aged under one year and suffering from postpartum depression | 15 female | Range: 17-19 Mean: 18.8 | To explore the experiences of teenage mothers with postpartum depression focussing on their experiences of being a teenage mother; support needs and the potential for support and education to be delivered by healthcare workers, or peers. |
| Bowman et al., 2015, US | Cross-sectional (Questionnaire) | 52 pregnant women taking antidepressants for anxiety and/or depression | 52 female | Range: 19-44 Mean: 39.04 | To determine the key influencing sources of information women receive or seek out that impact their decision to either continue or discontinue pharmacologic treatment for anxiety and/or depression during their pregnancy. |
| Bringewatt, 2013, US | Qualitative (Interview) | 15 emerging adults who were diagnosed with depression before the age of 17; 8 emerging adults who were diagnosed with generalised anxiety disorder before the age of 17 | N/A | Range: 18-22 | To examine the retrospective accounts of emerging adults who were diagnosed with mental health disorders in childhood to better understand how they made sense of their diagnoses over time. |
| Brydges et al., 2020, UK | Qualitative (Interview) | 14 men who were prescribed antidepressants to treat depression, including those treating depression with anxiety | 14 male | Range: 26-61 Mean: 49 | To explore views of men around their medication for the treatment of depression and the role of community pharmacy in their treatment. |
| Cartwright et al., 2016, New Zealand | Mixed method (Online survey) | 180 patients who were long-term users of antidepressants (3-15 years) | 143 female, 37 male | N/A | To examine patients’ views and experiences of long-term antidepressant treatment, including benefits and concerns |
| Clarke et al., 2018, UK | Qualitative (Analysis of emails) | 56 patients with depression; 10 patients with comorbid anxiety | N/A | N/A | To examine referral emails to describe the clinical characteristics of people who self-refer and explore the reasons for self-referral for transcranial magnetic stimulation treatment. |
| Dezetter et al., 2015, Canada | Longitudinal (Questionnaire) | 1288 adults with depressive and/or anxious symptomatology | 962 female, 326 male | Mean: 44.0 | To evaluate the mental health care needs perceived as unmet by adults in Quebec who had experienced depressive and/or anxious symptomatology in the previous 2 years and who used primary care services |
| Fakhoury, 2002, UK | Cross-sectional (Analysis of data collected by mental health helpline) | 658 sufferers of depression; 27 sufferers of anxiety | N/A | N/A | To describe the profile and experience with services of suicidal people calling SANELINE, a national mental health helpline in the UK. |
| Fonseca et al., 2016, Portugal | Cross-sectional (Online survey) | 131 women who were pregnant or had given birth during the last 12 months, and self-reported clinically significant depressive symptoms | 131 female | N/A | To characterise women’s current pattern of use of online resources for mental heal issues and women’s acceptance of e-mental health tools during the perinatal period, and to investigate its main determinants. |
| Gabriel & Violato, 2011, Canada | Cross-sectional (Questionnaire) | 63 outpatients with depression who were attending psychiatry clinic | 41 female, 22 male | Range: 19-65 Mean: 43.0 SD: 11.3 | To develop and psychometrically assess the knowledge seeking instrument (KSI) that can be easily used in clinical practice to measure knowledge-seeking behaviour in patients suffering from depression. |
| Garfield et al., 2004, UK | Prospective (Two qualitative interviews with a 3-month interval) | 51 patients with unipolar depression who had begun a new course of antidepressant medication in the previous 3 months | 29 female, 22 male | Range: 19-61 Mean: 41 | To identify information needs and the level of involvement in decision making desired by patients beginning courses of antidepressant medication, in order to inform the development of a concordant approach responsive to patients’ needs. |
| Glattacker et al., 2018, Germany | Sequential control group design (Questionnaire) | 205 patients diagnosed with a depressive episode or recurrent depressive disorder | 153 female, 52 male | Mean: 48.0 SD: 9.1 | To evaluate an intervention that applied the Common Sense Model to the provision of information during inpatient rehabilitation for patients with depression. |
| Graham et al., 2015, Australia | Cross-sectional (National survey) | 451 Australians who had a depressive episode in the 12 months before the survey | 300 female, 151 male | Mean: 41.0 | To explore the extent to which mental illness information is received by people with depression, its perceived helpfulness and to characterise those who do not receive such information. |
| Guillaumie et al., 2018, Canada | Qualitative (Interview) | 14 individuals diagnosed with major depression who had initiated antidepressant drug treatment at some point in the 12 months prior to their participation in the study | 11 female, 3 male | N/A | To explore patients’ experiences with the services community pharmacists provide for antidepressant drug treatment and identify potential avenues for improvement of pharmacists’ services within the context of antidepressant drug treatment. |
| Hallett et al., 2013, UK | Mixed-method (Survey with open- and closed-ended questions) | 96 patients with mood disorders who were receiving secondary mental health care and attending an outpatient clinic | 51 female, 45 male | N/A | To explore what outpatients want to learn about their illness and how they would like to learn this information. |
| Kalckreuth et al., 2014, Germany | Cross-sectional (Questionnaire) | 149 patients with affective disorders; 60 patients with neurotic, stress-related and somatoform disorders | N/A | N/A | To analyse the quantity and pattern of internet usage among mental health patients. |
| Kessing et al., 2006, Denmark | Cross-sectional (Questionnaire) | 258 patients with a diagnosis of depressive disorder | 171 female, 87 male | Median: 43.8 | To assess satisfaction in patients with depressive and bipolar disorders. |
| Khazaal et al., 2008, Switzerland | Cross-sectional (Questionnaire) | 149 patients treated in outpatient clinic for mood or anxiety disorders | N/A | N/A | To evaluate the use of internet by patients with psychiatric disorders in searching for general and medical information. |
| Kivelitz et al., 2018, Germany | Qualitative (Interview) | 16 patients with affective disorder; 3 patients with anxiety disorders | N/A | N/A | To investigate the information needs and the decision-making preferences of patients with mental disorders prior to the decision for a certain treatment setting. |
| Kronmüller et al., 2006, Germany | Cross-sectional (Questionnaire) | 56 patients with major depression | N/A | N/A | To identify psychosocial factors associated with greater or lesser knowledge about affective disorders in patients with depression. |
| Lewy et al., 2014, US | Cross-sectional (Online survey) | 569 military wives with varying degrees of depression | 569 female | Range: 18-56 Mean: 29.0 SD: 8.0 | To describe barriers to mental health care perceived by wives of military service members and to compare barriers for military wives with those experienced by similar women in the general population. |
| Liebherz et al., 2015a, Germany | Cross-sectional (Online survey) | 60 participants who self-reported experience with anxiety as a patient | 35 female, 25 male | Range: 18-62 Mean: 33.3 SD: 10.5 | To determine patients’ information and decision-making needs as a pre-requisite for the development of patient decision aids for anxiety disorders. |
| Liebherz et al., 2015b, Germany | Cross-sectional (Online survey) | 112 participants who self-reported experience with unipolar depression as a patient | 79 female, 32 male, 1 other | Range: 18-77 Mean: 42.0 SD: 12.7 | To describe patients’ information and decision-making needs as a prerequisite for the development of high-quality, web-based patient decision aids for affective disorders. |
| Llewellyn-Jones et al., 2001, UK | Qualitative (Interview) | 52 patients with unipolar depression; 14 patients with anxiety | N/A | N/A | To identify the questions patients most commonly ask their psychiatrist. |
| Louch et al., 2006, UK | Qualitative (Interview) | 9 patients with depression prescribed with antidepressants for no more than 9 months | 6 female, 3 male | Range: 29-62 Mean: 49.6 SD: 12.8 | To understand the experiences, expectations and needs of service users with mild to moderate depression, and to use these to inform the design and redesign of local services in primary care. |
| Maloni et al., 2013, US | Cross-sectional (Questionnaire) | 53 women between 2 weeks and 6 months postpartum who self-reported feelings of postpartum depression across the past week | 53 female | Range: 23-45 Mean: 32.6 SD: 5.0 | To determine women's barriers to treatment, use of online resources for assistance with postpartum depression, and preferences for internet treatment. |
| Mason & Francis, 2020, US | Cross-sectional (Questionnaire) | 128 Gulf Coast residents who experienced weather-related disasters (66% of participants reported a high frequency of depression and/or anxiety symptoms) | 112 female, 16 male | Range: 30-82 Mean: 50.5 SD: 12.9 | To understand factors related to weather-related disaster survivors’ health information and mental health-care-seeking behaviours. |
| McMenamy et al., 2008, US | Cross-sectional (Questionnaire) | 63 adult survivors of suicide (75% reported moderate to high levels of depression, 64% reported moderate to high levels of anxiety) | 45 female, 18 male | Mean: 50.5 SD: 9.9 | To examine the natural coping efforts of different suicide survivors through a newly developed survey instrument. |
| Moon et al., 2021, US | Qualitative (Analysis of Google search activity) | 37 patients with major depressive disorder who were hospitalised for suicidal thoughts and behaviours | N/A | N/A | To evaluate the internet search activity of suicidal young people to find evidence of suicidal ideation and behavioural health-related content. |
| Mueser et al., 1992, US | Cross-sectional (Questionnaire) | 14 patients with major affective disorder | N/A | N/A | To determine specific educational needs and to compare the needs of different consumers |
| Nestoriuc et al., 2021, Germany | Experimental (Online survey) | 97 patients taking recently prescribed antidepressants (≤4 months intake) | 55 female, 42 male | Mean: 39.1 SD: 11.9 | To investigate whether informing about the nocebo effect using a short information sheet affects patients’ need for information about antidepressants. |
| Nimrod, 2013, Israel | Cross-sectional (Online survey) | 793 members of 16 online depression communities (72% were diagnosed with major depression, 5% with dysthymia) | 555 female, 238 male | Range: 12-71 Mean: 36.0 | To explore whether members of online depression communities vary in their interests in issues discussed in the communities, and if so, whether groups with different interests also differ with regard to the benefits gained from participation. |
| Oh et al., 2020, US | Cross-sectional (Online survey) | 63 university students with self-reported depressive symptoms; 111 university students with self-reported anxiety symptoms | N/A | N/A | To examine university students' help-seeking behaviour, information needs, information-seeking behaviour, and information-seeking satisfaction when experiencing stress, anxiety, or depression symptoms. |
| Peden, 1994, US | Qualitative (Interview) | 7 women who had at one time been hospitalised with a diagnosis of depression and who considered themselves to be recovering at the time of this study | 7 female | Range: 29-53 Mean: 39 | To describe treatment strategies, interventions, and skills used by women to recover from depression. |
| Pohjanoksa-Mäntylä et al., 2009, Finland | Qualitative (Focus group) | 29 Internet users with present or past diagnosis of depression and use of an antidepressant | 26 female, 3 male | Range: 20-69 Mean: 47 | To assess how and why people use the Internet to access antidepressant information, and the self-reported impact of information obtained online. |
| Powell & Clarke, 2006, UK | Qualitative (Interview) | 16 participants with self-reported diagnosis of depression; 5 participants with self-reported diagnosis of anxiety disorder | Depression: 10 female, 6 male; Anxiety; 4 female, 1 male | N/A | To explore information needs and information seeking behaviour by mental health service users, to identify common themes, and to highlight areas that health services should target. |
| Quinn et al., 2014, Australia | Mixed method (Telephone interviews) | 23 individuals who had at least one first-degree relative with major depressive disorder, 91% of which also had personal experience of depression | 20 female, 3 male | Range: 23-64 Mean: 36.0 SD: 11.6 | To examine the information needs and preferences of people at increased familial risk for major depressive disorder, as well as the content, format and delivery of education targeted to people at increased familial risk. |
| Ramos-García et al., 2021, Spain | Cross-sectional (Online survey) | 70 participants with current or past diagnosis of generalised anxiety disorder | 47 female, 23 male | Mean: 41.09 SD = 12.21 | To assess patients’ information needs about generalised anxiety disorder. |
| Seedat et al., 2002, South Africa | Cross-sectional (Questionnaire) | 404 members of a depression and anxiety support group (39% of patient members with anxiety-only, 8% depression-only, 26% mixed anxiety-depression) | 320 female, 84 male | N/A | To measure the incidence of factors which might impact negatively on treatment-seeking, and to examine the relationship of patient and health care provider in the diagnosis and treatment of mental illness. |
| Simon et al., 2007, Germany | Qualitative (Interview) | 40 depressed patients who were already engaged in outpatient, inpatient and self-help group treatment for depression | 24 female, 16 male | Range: 18-70 Mean: 43.2 SD: 12.2 | To investigate depressed patients' perceptions of the treatment decision-making process with general practitioners. |
| Stacey et al., 2008, Canada | Mixed methods (Interview & Questionnaire) | 69 patients diagnosed with depression | N/A | N/A | To explore the decision-making needs of patients considering treatment options for their depression. |
| Stein et al., 2001, South Africa | Cross-sectional (Questionnaire) | 184 sufferers of anxiety disorders | N/A | N/A | To examine the role of consumer advocacy groups in psychiatry. |
| Tsai, 2006, Taiwan | Cross-sectional (Questionnaire) | 121 elderly nursing home residents with depressive symptoms | 71 female, 50 male | Mean: 79.1 SD: 6.7 | To explore self-care management strategies and risk factors for depressive symptoms among elderly residents of nursing homes in Taiwan. |
| Tsai, 2007, Taiwan | Cross-sectional (Questionnaire) | 98 elderly residents of public care homes with depressive symptoms | 32 female, 66 male | Mean: 79.7 SD: 7.1 | To determine the self-care strategies and risk factors for depressive symptoms among residents of public elder care homes in Taiwan. |
| Tsai et al., 2012, Taiwan | Cross-sectional (Questionnaire) | 172 elderly outpatients with depressive symptoms | 102 female, 70 male | Mean: 73.7 SD: 6.4 | To explore self-care management strategies and risk factors for depressive symptoms among elderly outpatients in Taiwan. |
| Uebelacker et al., 2012, US | Qualitative (Focus group) | 30 Latino participants who endorsed having been depressed themselves or having had a close friend or family member with depression, stress, nervios, or worries | 26 female, 3 male, 1 unknown | N/A | To ask about the barriers to and facilitators of depression treatment in general as well as barriers to participation in depression telephone care management. |
| van Geffen et al., 2009, Netherlands | Qualitative (Interview) | 41 patients who had recently started a new course of selective serotonin-reuptake inhibitor treatment (61.0% used the medication for depression, 17.1% for anxiety, and 14.6% for nonspecific mood symptoms) | 25 female, 16 male | N/A | To examine patients’ perceptions of information received at the start of selective serotonin-reuptake inhibitor treatment, aiming to identify information needs and the potential role of the community pharmacist as information provider. |
| Van Meter et al., 2019, US | Cross-sectional (Questionnaire) | 30 patients with depression; 5 patients with generalised anxiety disorder | N/A | N/A | To investigate the online help- and information-seeking activity of young people newly diagnosed with mood and anxiety disorders in order to better understand how digital resources might serve this population. |
| Webelhorst et al., 2020, Germany | Cross-sectional (Questionnaire) | 158 patients with affective disorders; 64 patients with neurotic, stress-related, and somatoform disorders | N/A | N/A | To examine how web-based services currently influence mental health care, asking about previous internet use and future interests of patients with mental disorders. |
| Zaini et al., 2018, Malaysia | Qualitative (Focus group) | 11 patients diagnosed with major depressive disorder | 10 female, 1 male | N/A | To develop a strategic tool towards the promotion and implementation of shared decision-making in the use of antidepressants among patients with major depressive disorder. |
| Zapata, et al., 2018, US | Mixed-method (Interview & Questionnaire) | 11 veterans with high anxiety symptoms | 3 female, 8 male | Mean: 66.73 SD = 5.78 | To understand where older veterans seek information about anxiety and coping. |

Table S3. *Information needs reported by studies on people with depression (k = 46 studies).*

| Theme | Information needs | Number and percentage of studies | References |
| --- | --- | --- | --- |
| General facts about depression (k = 32; 69.6%) | Symptoms/signs of depression | 15 (46.9%) | (1–15) |
|  | General information on depression | 13 (40.6%) | (2,4–7,14,16–22) |
|  | Diagnosis (diagnostic criteria; meaning of diagnosis) | 11 (34.4%) | (1,3,11,23–30) |
|  | Aetiology (causes of depression; scientific details) | 10 (31.3%) | (3,5,7,11,12,14,24,26,31,32) |
|  | Prognosis (length/course of depression; recovery) | 6 (18.8%) | (5,13,21,26,27,31) |
|  | Information on suicidal thoughts | 4 (12.5%) | (5,12,15,33) |
|  | Prevalence of depression | 3 (9.4%) | (3,7,11) |
|  | Recent research on depression | 2 (6.3%) | (11,14) |
|  | Whether depression is normal | 2 (6.3%) | (3,29) |
|  | Environmental risk factors (e.g., stress, lifestyle) | 2 (6.3%) | (11,14) |
|  | Risks of developing depression based on family history | 2 (6.3%) | (11,14) |
|  | Behavioural problems (e.g., violence, drug/alcohol abuse) | 1 (3.1%) | (11) |
| Treatment (k = 38; 82.6%) | Treatment options and comparison between options | 24 (63.2%) | (1,4,19,23,24,26,28–32,34,5,35–38,6,7,11–13,15,17) |
|  | Side effects of treatment | 19 (50.0%) | (1,3,24,28–32,38–40,5,7,9,11,18,19,21,23) |
|  | Effectiveness/benefits of treatment and expected outcomes | 15 (39.5%) | (5,7,35–37,41,42,9,19,21,24,29–32) |
|  | General information on medication | 14 (36.8%) | (1,3,26,36,41,43,5–7,11,12,19,22,25) |
|  | Appropriate use of medication (e.g., dosage, long-term use, discontinuation) | 13 (34.2%) | (5,12,42,44,45,21,24,32,36,38–41) |
|  | Length of treatment | 7 (18.4%) | (19,21,29,32,35,42,44) |
|  | Explanation of specific procedures and approaches used | 4 (10.5%) | (27,30,32,41) |
|  | Issues related to addiction, tolerance, and dependence of medication | 4 (10.5%) | (24,32,39,42) |
|  | Adverse drug reactions | 4 (10.5%) | (32,36,42,44) |
|  | Psychosocial or nonpharmacological interventions | 3 (7.9%) | (4,21,43) |
|  | Mechanisms or (non-)pharmacological actions of medications | 2 (5.3%) | (32,40) |
|  | Placebo/nocebo effects | 1 (2.6%) | (40) |
|  | What does the prescribed dose indicate about patients' condition | 1 (2.6%) | (44) |
| Lived experience (k = 15; 32.6%) | Other people's experience of depression in general | 10 (66.7%) | (2,4,6,7,17,26,30,31,35,38) |
|  | Other people's experience of taking medications for depression | 4 (26.7%) | (19,24,32,36) |
|  | Other people's experience of recovery from depression | 2 (13.3%) | (24,30) |
|  | Other people's experience of antidepressant withdrawal and depression relapse | 1 (6.7%) | (39) |
|  | Other people's reasons to use antidepressants | 1 (6.7%) | (36) |
|  | Previous patients' experience of treatment | 1 (6.7%) | (38) |
| Healthcare services (k = 24; 52.2%) | Available local services (e.g., hospitals, day treatment, rehabilitation) | 14 (58.3%) | (1,2,28,31,33,35,4–7,11,22,25,27) |
|  | How or where to get help | 8 (33.3%) | (16,18,23,24,33,35,43,46) |
|  | Support groups & patient associations | 7 (29.2%) | (2,5,22,25,26,28,33) |
|  | Healthcare professionals (e.g., psychologists, psychiatrists) | 6 (25.0%) | (4–6,22,31,33) |
|  | Information on the mental health system | 2 (8.3%) | (11,24) |
|  | Role of psychologists | 2 (8.3%) | (5,29) |
|  | How to get a further supply of medication | 1 (4.2%) | (21) |
| Coping & self-management (k = 17; 37.0%) | Strategies to cope with depression and alleviate symptoms | 13 (76.5%) | (1,2,24,26,31,3,4,7,11–13,16,17) |
|  | Improving independent living skills (coping with everyday life) | 5 (29.4%) | (11,12,17,31,32) |
|  | Managing medication side effects | 4 (23.5%) | (21,32,36,47) |
|  | Improving social relationships and communication skills | 3 (17.6%) | (11,13,24) |
|  | Coping with stigma/discrimination | 3 (17.6%) | (7,11,24) |
|  | Strategies to speed recovery or prevent exacerbation | 2 (11.8%) | (21,26) |
|  | Coping with unpredictable variations in intensity and duration of depression | 1 (5.9%) | (24) |
|  | Strategies for solving problems | 1 (5.9%) | (11) |
|  | Dealing with weight gain | 1 (5.9%) | (11) |
|  | What to do in case of no response to medication | 1 (5.9%) | (32) |
| Financial & legal information (k = 8; 17.4%) | Financial assistance | 7 (87.5%) | (11,18,25,26,29,35,36) |
|  | Cost of treatment | 2 (25.0%) | (7,36) |
|  | Mental health law | 1 (12.5%) | (25) |
| Other information needs (k = 9; 19.6%) | Current health condition and other comorbid health problems | 2 (22.2%) | (19,24) |
|  | Social relationships and social isolation/avoidance/withdrawal | 2 (22.2%) | (11,12) |
|  | How to figure out severity of mental disorder | 2 (22.2%) | (7,24) |
|  | Interpretation of information from patient information leaflets and the internet | 1 (11.1%) | (44) |
|  | What to do between visits | 1 (11.1%) | (27) |
|  | Information for relatives | 1 (11.1%) | (31) |
|  | Work-related challenges | 1 (11.1%) | (12) |
|  | How to reduce the likelihood of depression for themselves and their family | 1 (11.1%) | (14) |

Table S4. *Information needs reported by studies on people with anxiety (k = 23 studies).*

| Theme | Information Needs | Number and Percentage of Studies | References |
| --- | --- | --- | --- |
| General facts about anxiety (k = 17; 73.9%) | Symptoms | 9 (52.9%) | (1,3,6–8,10,15,48,49) |
|  | Diagnosis (diagnostic criteria; meaning of diagnosis) | 7 (41.2%) | (1,3,23,25,28,30,48) |
|  | Aetiology (causes of anxiety; scientific details) | 5 (29.4%) | (3,7,8,32,48) |
|  | General information on anxiety | 3 (17.6%) | (6,7,22) |
|  | Prevalence of anxiety | 3 (17.6%) | (3,7,29) |
|  | Prognosis (length/course of anxiety; recovery) | 3 (17.6%) | (8,29,48) |
|  | Whether anxiety is normal | 2 (11.8%) | (3,29) |
|  | Information on suicidal thoughts | 2 (11.8%) | (15,33) |
|  | Risks of developing anxiety based on family history | 1 (5.9%) | (48) |
| Treatment (k = 21; 91.3%) | Treatment options and comparison between options | 12 (57.1%) | (1,6,35,48,7,8,15,28–30,32,34) |
|  | Side effects of treatment | 11 (52.4%) | (1,3,49,7,8,23,28,30,32,40,48) |
|  | General information on medication | 9 (42.9%) | (1,3,6–8,22,25,43,49) |
|  | Effectiveness/benefits of treatment and expected outcomes | 7 (33.3%) | (7,8,30,32,35,48,49) |
|  | Length of treatment | 4 (19.0%) | (29,32,35,44) |
|  | Psychosocial or nonpharmacological interventions | 4 (19.0%) | (7,8,43,49) |
|  | Appropriate use of medication (e.g., dosage) | 4 (19.0%) | (32,40,44,45) |
|  | Mechanisms or (non-)pharmacological actions of medications | 3 (14.3%) | (32,40,48) |
|  | Explanation of specific procedures and approaches used | 2 (9.5%) | (30,32) |
|  | Issues related to addiction, tolerance, and dependence of medication | 2 (9.5%) | (32,48) |
|  | Adverse drug reactions | 2 (9.5%) | (32,44) |
|  | What does the prescribed dose indicate about patients' condition | 1 (4.8%) | (44) |
|  | Placebo/nocebo effects | 1 (4.8%) | (40) |
| Lived experience (k = 7; 30.4%) | Other people's experience of anxiety in general | 6 (85.7%) | (6–8,30,35,48) |
|  | Other people's experience of taking medications for anxiety | 1 (14.3%) | (32) |
|  | Other people's experience of recovery from anxiety | 1 (14.3%) | (30) |
| Healthcare services (k = 14; 60.9%) | Available local services (e.g., hospitals, clinics, day care) | 11 (78.6%) | (1,6,49,7,8,22,25,28,29,35,48) |
|  | Healthcare professionals (e.g., psychologists, psychiatrists) | 6 (42.9%) | (6,8,22,33,48,49) |
|  | How or where to get help | 5 (35.7%) | (23,33,35,43,48) |
|  | Support groups & patient associations | 5 (35.7%) | (22,25,28,33,48) |
|  | Role of psychologists | 1 (7.1%) | (29) |
| Coping & self-management (k = 8; 34.8%) | Strategies to cope with anxiety and alleviate symptoms | 7 (87.5%) | (1,3,7,8,29,48,49) |
|  | Improving independent living skills (coping with everyday life) | 3 (37.5%) | (8,32,48) |
|  | Managing medication side effects | 1 (12.5%) | (32) |
|  | What to do in case of no response to medication | 1 (12.5%) | (32) |
|  | Strategies to improve self-esteem | 1 (12.5%) | (48) |
|  | Coping with stigma/discrimination | 1 (12.5%) | (7) |
| Financial & legal information (k = 3; 13.0%) | Financial assistance | 2 (66.7%) | (25,35) |
|  | Cost of treatment | 1 (33.3%) | (7) |
|  | Mental health law | 1 (33.3%) | (25) |
| Other information needs (k = 4; 17.4%) | Information for relatives | 2 (50.0%) | (8,48) |
|  | How to figure out severity of mental disorder | 1 (25.0%) | (7) |
|  | Interpretation of information from patient information leaflets and the internet | 1 (25.0%) | (44) |

Table S5. *Current information sources reported by studies on people with depression (k = 44 studies).*

| Categories | Subcategories | Number and percentage of studies | References |
| --- | --- | --- | --- |
| Health professionals (k = 31, 70.5%) | General healthcare professionals (e.g., general practitioners, doctors, physicians) | 26 (83.9%) | (7,9,10,13–15,17–19,23,26,30–32,35,36,38–41,44,45,50–53) |
|  | Mental health professionals (e.g., psychiatrists, psychologists, counsellors) | 12 (38.7%) | (3,7,41,45,19,23,26,29,31,33,35,40) |
|  | Pharmacists | 6 (19.4%) | (32,36,41,44,45,47) |
|  | Email physicians | 1 (3.2%) | (36) |
|  | Email, chat, or online appointments with mental health professionals | 1 (3.2%) | (4) |
| Written materials (k = 21, 47.7%) | Patient information leaflets, medicine labels, brochures, and pamphlets | 13 (61.9%) | (9,18,50,54,55,19,26,30,32,36,40,41,44) |
|  | Books (e.g., fiction, nonfiction, self-help books, textbooks) | 12 (57.1%) | (3,7,54,55,13,14,17,19,26,30,33,50) |
|  | Newspapers and magazines | 7 (33.3%) | (17,23,26,34,40,50,55) |
| Media (k = 32, 72.7%) | Internet (e.g., depression websites, search engines, Wikipedia) | 27 (84.4%) | (1,3,17–19,22,26,28,30–33,4,34,36,40,44,45,54,55,5–7,10,14–16) |
|  | Broadcast media (e.g., television, videos, commercials, radio, and CDs) | 11 (34.4%) | (3,17,55,18,19,23,26,40,41,50,54) |
|  | Online forums, online support groups, blogs, and social media | 8 (25.0%) | (4,6,7,12,22,24,36,45) |
| Interpersonal (k = 20, 45.5%) | Talk to friends and relatives | 15 (75.0%) | (3,7,45,50–53,10,15,17,19,26,36,40,41) |
|  | Self-help or support groups | 6 (30.0%) | (2,23,26,30,33,49) |
|  | Email friends | 1 (5.0%) | (36) |
|  | Minister or spiritual advisors | 1 (5.0%) | (7) |
| Organisational (k = 8, 18.2%) | Mental health organisations (e.g., advocacy organisations, substance abuse services, elder services) | 3 (37.5%) | (26,30,33) |
|  | General healthcare system and public sources | 2 (25.0%) | (17,19) |
|  | Telephone services and hotlines | 2 (25.0%) | (33,36) |
|  | Classes on mental health (e.g., stress management) | 2 (25.0%) | (3,13) |

Table S6. *Current information sources reported by studies on people with anxiety (k = 22 studies).*

| Categories | Subcategories | Number and percentage of studies | References |
| --- | --- | --- | --- |
| Health professionals (k = 16, 72.7%) | General healthcare professionals (e.g., general practitioners, doctors, physicians) | 13 (81.3%) | (7,8,45,49,56,10,15,23,30,32,35,40,44) |
|  | Mental health professionals (e.g., psychiatrists, psychologists, counsellors) | 10 (62.5%) | (3,7,8,23,29,33,35,40,45,49) |
|  | Pharmacists | 3 (18.8%) | (32,44,45) |
| Written materials (k = 11, 55.0%) | Patient information leaflets, medicine labels, brochures, and pamphlets | 5 (45.5%) | (30,32,40,44,49) |
|  | Newspapers and magazines | 5 (45.5%) | (23,34,40,49,56) |
|  | Books (e.g., self-help books, textbooks) | 5 (45.5%) | (3,7,30,33,56) |
| Media (k = 19, 86.4%) | Internet (e.g., anxiety disorder websites, search engines, Wikipedia) | 18 (94.7%) | (1,3,32–34,40,44,45,48,56,6–8,10,15,22,28,30) |
|  | Broadcast media (e.g., television, videos, commercials, radio, and CDs) | 4 (21.1%) | (3,23,40,56) |
|  | Online forums, online support groups, blogs, and social media | 4 (21.1%) | (6,7,22,45) |
| Interpersonal (k = 11, 50.0%) | Friends and relatives | 7 (63.6%) | (3,7,10,15,40,45,56) |
|  | Self-help or support groups | 5 (45.5%) | (23,30,33,40,49) |
|  | Minister or spiritual advisors | 2 (18.2%) | (7,49) |
| Organisational (k = 4, 18.2%) | Mental health organisations (e.g., advocacy organisations, substance abuse services, elder services) | 2 (50.0%) | (30,33) |
|  | General healthcare system and public sources | 1 (25.0%) | (56) |
|  | Telephone services and hotlines | 1 (25.0%) | (33) |
|  | Classes on mental health (e.g., stress management) | 1 (25.0%) | (3) |

Table S7. *Preferred information sources reported by studies on people with depression (k = 13 studies).*

| Categories | Subcategories | Number and percentage of studies | References |
| --- | --- | --- | --- |
| Health professionals (k = 10, 76.9%) | General healthcare professionals (e.g., general practitioners, doctors, physicians) | 8 (80.0%) | (14,15,19,20,26,36,38,39) |
|  | Mental health professionals (e.g., psychiatrists, psychologists, counsellors) | 2 (20.0%) | (14,26) |
|  | Pharmacists | 2 (20.0%) | (36,38) |
|  | Email pharmacists | 1 (10.0%) | (32) |
|  | Chat or online appointments with depression experts | 1 (10.0%) | (16) |
| Written materials (k=6, 46.2%) | Patient information leaflets, brochures, and pamphlets | 4 (66.7%) | (2,18,26,37) |
|  | Books (e.g., literature on depression) | 4 (66.7%) | (20,26,36,37) |
|  | Magazines | 2 (33.3%) | (20,37) |
| Media (k = 9, 69.2%) | Broadcast media (e.g., television, videos, commercials, radio, and CDs) | 7 (77.8%) | (2,14,16,18,20,26,37) |
|  | Internet (e.g., depression websites, search engines, Wikipedia) | 6 (66.7%) | (2,14,15,26,32,37) |
|  | Online forums, online support groups, blogs, and social media | 1 (11.1%) | (16) |
| Interpersonal (k = 2, 15.4%) | Friends and relatives | 2 (100.0%) | (15,26) |
|  | Self-help or support groups | 1 (50.0%) | (26) |
| Organisational (k = 4, 30.8%) | Educational outreach in hospitals, schools, and churches | 3 (75.0%) | (15,18,26) |
|  | Telephone services and hotlines | 1 (25.0%) | (36) |
|  | Informative signs or tables at community events | 1 (25.0%) | (18) |

**References**

1. Balhara YPS, Singh S, Bhargava R. The pattern of problematic internet use and mental health-related internet use among psychiatric outpatients at a tertiary care center. Tzu Chi Med J. 2020;32(2):198–204.

2. Boath EH, Henshaw C, Bradley E. Meeting the challenges of teenage mothers with postpartum depression: overcoming stigma through support. J Reprod Infant Psychol. 2013;31(4):352–69.

3. Bringewatt EH. Negotiating narratives surrounding children’s mental health diagnoses: Children and their contribution to the discourse. Child Youth Serv Rev [Internet]. 2013;35:1219–26. Available from: http://dx.doi.org/10.1016/j.childyouth.2013.04.008

4. Fonseca A, Gorayeb R, Canavarro MC. Women’s use of online resources and acceptance of e-mental health tools during the perinatal period. Int J Med Inform [Internet]. 2016;94:228–36. Available from: http://dx.doi.org/10.1016/j.ijmedinf.2016.07.016

5. Moon KC, Van Meter AR, Kirschenbaum MA, Ali A, Kane JM, Birnbaum ML. Internet Search Activity of Young People with Mood Disorders Who Are Hospitalized for Suicidal Thoughts and Behaviors: Qualitative Study of Google Search Activity. JMIR Ment Heal. 2021;8(10):e28262.

6. Webelhorst C, Jepsen L, Rummel-Kluge C. Utilization of e-mental-health and online self-management interventions of patients with mental disorders-A cross-sectional analysis. PLoS One [Internet]. 2020;15(4):e0231373. Available from: http://dx.doi.org/10.1371/journal.pone.0231373

7. Oh CY, Kornfield R, Lattie EG, Mohr DC, Reddy M. University students’ information behavior when experiencing mental health symptoms. Proc Assoc Inf Sci Technol. 2020;57:e417.

8. Liebherz S, Härter M, Dirmaier J, Tlach L. Information and Decision-Making Needs Among People with Anxiety Disorders: Results of an Online Survey. Patient. 2015;8(6):531–9.

9. Louch P, Goodman C, Greenhalgh T. Involving service users in the evaluation and redesign of primary care services for depression: A qualitative study. Prim Care Community Psychiatry. 2006;10(3):109–17.

10. Mason NF, Francis DB. Information-Seeking and Use of Primary Care Mental Health Services among Gulf Coast Survivors of Natural Disasters. Disaster Med Public Health Prep. 2020;1–6.

11. Mueser KT, Bellack AS, Wade JH, Sayers SL, Rosenthal CK. An assessment of the educational needs of chronic psychiatric patients and their relatives. Br J Psychiatry. 1992;160:674–80.

12. Nimrod G. Online Depression Communities: Members’ Interests and Perceived Benefits. Health Commun. 2013;28:425–34.

13. Peden AR. Up from depression: strategies used by women recovering from depression. J Psychiatr Ment Health Nurs. 1994;1:77–83.

14. Quinn V, Meiser B, Wilde A, Cousins Z, Barlow-Stewart K, Mitchell PB, et al. Preferences regarding targeted education and risk assessment in people with a family history of major depressive disorder. J Genet Couns. 2014;23(5):785–95.

15. Van Meter AR, Birnbaum ML, Rizvi A, Kane JM. Online help-seeking prior to diagnosis: Can web-based resources reduce the duration of untreated mood disorders in young people? J Affect Disord [Internet]. 2019;252:130–4. Available from: https://doi.org/10.1016/j.jad.2019.04.019

16. Maloni JA, Przeworski A, Damato EG. Web Recruitment and Internet Use and Preferences Reported by Women With Postpartum Depression After Pregnancy Complications. Arch Psychiatr Nurs [Internet]. 2013;27:90–5. Available from: http://dx.doi.org/10.1016/j.apnu.2012.12.001

17. Simon D, Loh A, Wills CE, Härter M. Depressed patients’ perceptions of depression treatment decision-making. Heal Expect. 2007;10:62–74.

18. Uebelacker LA, Marootian BA, Pirraglia PA, Primack J, Tigue PM, Haggarty R, et al. Barriers and facilitators of treatment for depression in a Latino community: A focus group study. Community Ment Health J. 2012;48:114–26.

19. Anderson C, Roy T. Patient experiences of taking antidepressants for depression: A secondary qualitative analysis. Res Soc Adm Pharm [Internet]. 2013;9:884–902. Available from: http://dx.doi.org/10.1016/j.sapharm.2012.11.002

20. Boath EH, Bradley E, Anthony P. User’s views of two alternative approaches to the treatment of postnatal depression. J Reprod Infant Psychol. 2004;22(1):13–24.

21. Glattacker M, Heyduck K, Meffert C, Jakob T. Illness Beliefs, Treatment Beliefs and Information Needs as Starting Points for Patient Information: The Evaluation of an Intervention for Patients with Depression. J Clin Psychol Med Settings [Internet]. 2018;25(3):316–33. Available from: http://dx.doi.org/10.1007/s10880-018-9551-1

22. Kalckreuth S, Trefflich F, Rummel-Kluge C. Mental health related Internet use among psychiatric patients: A cross-sectional analysis. BMC Psychiatry. 2014;14:368.

23. Seedat S, Stein DJ, Berk M, Wilson Z. Barriers to treatment among members of a mental health advocacy group in South Africa. Soc Psychiatry Psychiatr Epidemiol. 2002;37:483–7.

24. Barney LJ, Griffiths KM, Banfield MA. Explicit and implicit information needs of people with depression: a qualitative investigation of problems reported on an online depression support forum. BMC Psychiatry. 2011;11:88.

25. Fakhoury WKH. Suicidal callers to a national helpline in the UK: A comparison of depressive and psychotic sufferers. Arch Suicide Res. 2002;6:363–71.

26. Hallett C, Gupta S, Priebe S. What do outpatients with schizophrenia and mood disorders want to learn about their illness? Psychiatr Serv. 2013;64(8):764–9.

27. Kessing LV, Hansen HV, Ruggeri M, Bech P. Satisfaction with treatment among patients with depressive and bipolar disorders. Soc Psychiatry Psychiatr Epidemiol. 2006;41:148–55.

28. Khazaal Y, Chatton A, Cochand S, Hoch A, Khankarli MB, Khan R, et al. Internet use by patients with psychiatric disorders in search for general and medical informations. Psychiatr Q. 2008;79:301–9.

29. Llewellyn-Jones S, Jones G, Donnelly P. Questions patients ask psychiatrists. Psychiatr Bull. 2001;25:21–4.

30. Powell J, Clarke A. Information in mental health: qualitative study of mental health service users. Heal Expect. 2006;9:359–65.

31. Liebherz S, Tlach L, Härter M, Dirmaier J. Information and decision-making needs among people with affective disorders – results of an online survey. Patient Prefer Adherence. 2015;9:627–38.

32. Van Geffen ECG, Kruijtbosch M, Egberts ACG, Heerdink ER, Van Hulten R. Patients’ perceptions of information received at the start of selective serotonin-reuptake inhibitor treatment: Implications for community pharmacy. Ann Pharmacother. 2009;43:642–9.

33. Mcmenamy JM, Jordan JR, Mitchell AM. What do Suicide Survivors Tell Us They Need? Results of a Pilot Study. Suicide Life-Threatening Behav. 2008;38(4):375–89.

34. Clarke M, Lankappa S, Burnett M, Khalifa N, Beer C. Patients with depression who self-refer for transcranial magnetic stimulation treatment: exploratory qualitative study. BJPsych Bull. 2018;42(6):243–7.

35. Kivelitz L, Härter M, Mohr J, Melchior H, Goetzmann L, Warnke MH, et al. Choosing the appropriate treatment setting: Which information and decision-making needs do adult inpatients with mental disorders have? A qualitative interview study. Patient Prefer Adherence. 2018;12:823–33.

36. Pohjanoksa-Mäntylä M, Saari JK, Närhi U, Karjalainen A, Pylkkänen K, Airaksinen MS, et al. How and why do people with depression access and utilize online drug information: A qualitative study. J Affect Disord [Internet]. 2009;114:333–9. Available from: http://dx.doi.org/10.1016/j.jad.2008.06.024

37. Stacey D, Menard P, Gaboury I, Jacobsen M, Sharif F, Ritchie L, et al. Decision-making needs of patients with depression: a descriptive study. J Psychiatr Ment Health Nurs. 2008;15:287–95.

38. Zaini S, Bharathy HAM, Sulaiman AH, Gill JS, Hui KO, Huri HZ, et al. Development of a strategic tool for shared decision-making in the use of antidepressants among patients with major depressive disorder: A focus group study. Int J Environ Res Public Health. 2018;15:1402.

39. Cartwright C, Gibson K, Read J, Cowan O, Dehar T. Long-term antidepressant use: Patient perspectives of benefits and adverse effects. Patient Prefer Adherence. 2016;10:1401–7.

40. Nestoriuc Y, Pan Y, Kinitz T, Weik E, Shedden-Mora MC. Informing About the Nocebo Effect Affects Patients’ Need for Information About Antidepressants—An Experimental Online Study. Front Psychiatry. 2021;12:587122.

41. Al-Saffar N, Abdulkareem A, Abdulhakeem A, Salah AQ, Heba M. Depressed patients’ preferences for education about medications by pharmacists in Kuwait. Patient Educ Couns. 2008;72:94–101.

42. Garfield S, Francis S-A, Smith FJ. Building concordant relationships with patients starting antidepressant medication. Patient Educ Couns. 2004;55:241–6.

43. Dezetter A, Duhoux A, Menear M, Roberge P, Chartrand E, Fournier L. Reasons and Determinants for Perceiving Unmet Needs for Mental Health in Primary Care in Quebec. Can J Psychiatry. 2015;60(6):284–93.

44. Brydges S, Rennick-Egglestone S, Anderson C. Men’s views of antidepressant treatment for depression, and their implications for community pharmacy practice. Res Soc Adm Pharm. 2020;16:1041–9.

45. Bowman JA, Hellier SD, Cline TW. Sources Impacting Pharmacological Treatment for Anxiety and/or Depression During Pregnancy. J Nurse Pract [Internet]. 2015;11(2):184–91. Available from: http://dx.doi.org/10.1016/j.nurpra.2014.08.013

46. Lewy CS, Oliver CM, McFarland BH. Barriers to mental health treatment for military wives. Psychiatr Serv. 2014;65(9):1170–3.

47. Guillaumie L, Ndayizigiye A, Beaucage C, Moisan J, Grégoire JP, Villeneuve D, et al. Patient perspectives on the role of community pharmacists for antidepressant treatment: A qualitative study. Can Pharm J. 2018;151:142–8.

48. Ramos-García V, Rivero-Santana A, Duarte-Díaz A, Perestelo-Pérez L, Peñate-Castro W, Álvarez-Pérez Y, et al. Shared decision-making and information needs among people with generalized anxiety disorder. Eur J Investig Heal Psychol Educ. 2021;11:423–35.

49. Stein DJ, Wessels C, Zungu-Dirwayi N, Berk M, Wilson Z. Value and effectiveness of consumer advocacy groups: A survey of the anxiety disorders support group in South Africa. Depress Anxiety. 2001;13:105–7.

50. Kronmüller K-T, Saha R, Karr M, Kratz B, Hunt A, Mundt C, et al. Psychosocial factors associated with knowledge about affective disorders in patients with depression. Psychopathology. 2006;39:105–12.

51. Tsai Y-F. Self-Care Management and Risk Factors for Depressive Symptoms Among Elderly Nursing Home Residents in Taiwan. J Pain Symptom Manage. 2006;32(2):140–7.

52. Tsai Y-F. Self-care management and risk factors for depressive symptoms among Taiwanese institutionalized older persons. Nurs Res. 2007;56(2):124–31.

53. Tsai Y-F, Liu L-L, Tsai H-H, Chung S-C. Self-care management and risk factors for depressive symptoms among elderly outpatients in Taiwan. Int Psychogeriatrics. 2012;24(2):278–87.

54. Gabriel A, Violato C. Psychoeducational methods for patients suffering from depression: The knowledge seeking instrument (KSI). J Affect Disord [Internet]. 2011;133:406–12. Available from: http://dx.doi.org/10.1016/j.jad.2011.04.058

55. Graham AL, Hasking P, Clarke D, Meadows G. How People with Depression Receive and Perceive Mental Illness Information: Findings from the Australian National Survey of Mental Health and Wellbeing. Community Ment Health J. 2015;51:994–1001.

56. Zapata AML, Beaudreau SA, O’Hara R, Bereknyei Merrell S, Bruce J, Garrison-Diehn C, et al. Information-Seeking about Anxiety and Perceptions about Technology to Teach Coping Skills in Older Veterans. Clin Gerontol. 2018;41(4):346–56.
